# Linker Engineering of Ligand‐Decorated DNA Origami Nanostructures Affects Biological Activity

**DOI:** 10.1002/smll.202202704

**Published:** 2022-08-07

**Authors:** Carmen M. Domínguez, Miguel García‐Chamé, Ulrike Müller, Andreas Kraus, Klavdiya Gordiyenko, Ahmad Itani, Heiko Haschke, Peter Lanzerstorfer, Kersten S. Rabe, Christof M. Niemeyer

**Affiliations:** ^1^ Institute for Biological Interfaces (IBG‐1) Karlsruhe Institute of Technology (KIT) 76344 Eggenstein‐Leopoldshafen Germany; ^2^ School of Engineering University of Applied Sciences Upper Austria Wels 4600 Austria; ^3^ Bruker Nano GmbH JPK BioAFM, Am Studio 2D 12489 Berlin Germany

**Keywords:** cell signaling, DNA nanostructures, epidermal growth factor receptor (EGFR), Grb2, high‐speed AFM, receptor clustering, streptavidin

## Abstract

News from an old acquaintance: The streptavidin (STV)‐biotin binding system is frequently used for the decoration of DNA origami nanostructures (DON) to study biological systems. Here, a surprisingly high dynamic of the STV/DON interaction is reported, which is affected by the structure of the DNA linker system. Analysis of different mono‐ or bi‐dentate linker architectures on DON with a novel high‐speed atomic force microscope (HS‐AFM) enabling acquisition times as short as 50 ms per frame gave detailed insights into the dynamics of the DON/STV interaction, revealing dwell times in the sub‐100 millisecond range. The linker systems are also used to present biotinylated epidermal growth factor on DON to study the activation of the epidermal growth factor receptor signaling cascade in HeLa cells. The studies confirm that cellular activation correlated with the binding properties of linker‐specific STV/DON interactions observed by HS‐AFM. This work sheds more light on the commonly used STV/DON system and will help to further standardize the use of DNA nanostructures for the study of biological processes.

## Graphical abstract

DNA origami nanostructures (DON) decorated with bioactive ligands via streptavidin‐biotin bridges exhibit surprisingly high binding dynamics that are influenced by the underlying linker architecture. The results of binding studies obtained by high‐speed atomic force microscopy correlate with biological activity.

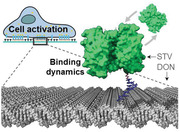

## 1 Introduction

In recent years, scaffolded DNA origami technology has emerged as a promising tool for studying biological processes, such as cell adhesion and activation.^[^ ^1^ ^]^ In these applications, DNA origami nanostructures (DON) are commonly used as molecular pegboards for the presentation of immobilized bioactive ligands to cells and tissues, thereby achieving complete control over the number and spatial arrangement of ligands at the lower nanoscale. However, close control of the synthesis and detailed characterization of ligand‐decorated DON is essential to exploit the potential of this approach, as surface occupancy and stability of DON‐ligand binding may represent crucial parameters for biological functionality. Therefore, while the preparation of pure DON is a highly straightforward and standardized technique, the functionalization of such nanostructures with protein ligands still poses a challenge. For example, the biotin‐streptavidin (STV) binding system is by far the most commonly used coupling system to build protein‐decorated DON,^[^ ^2^ ^]^ and the use of biotin‐STV bridges remains of dominant importance for the construction of DON to study biological systems.^[^ ^1b–d,f–j^ ^]^ and protein‐DON interactions.^[^ ^3^ ^]^ Nevertheless, stable occupancy densities of more than 75%–80% can hardly be achieved even with this robust method,^[^ ^1^, ^4^ ^]^ and this phenomenon has been attributed to the effectiveness of staple labeling and incorporation^[^ ^5^ ^]^ and the destruction of protein binding by the scanning tip during characterization by AFM (atomic force microscopy).^[^ ^6^ ^]^ Despite its enormously high affinity (*K*d ≈ 10^–13^ M) and long lifetime (τ ≈ 10 d, *k*_off_ ≈ 10^–6^ s^–1^), the STV/biotin complex is also subject to an unbinding process, of which an atomistic dynamic picture has recently been obtained using force spectroscopy and atomistic simulations.^[^ ^7^ ^]^ However, it remains unclear whether unbinding also affects the decoration of DNA nanostructures.

We investigate here to what extent the binding of STV to biotinylated staples on the surface of DON is influenced by the steric accessibility of the biotin residue and whether this also influences the biological activity of the DON structures. To this end, we studied the effects of three different, mono‐ or bi‐dentate linker systems on DON on the binding and dynamics of STV using high‐speed atomic force microscopy (HS‐AFM). In addition, these linker systems were used for the presentation of biotinylated epidermal growth factor (bioEGF) on DON and the effect of the ligand‐decorated DON on the activation of the epidermal growth factor receptor (EGFR) signaling cascade in HeLa cells was examined. Due to the high spatiotemporal resolution of HS‐AFM, we observed, for the first time surprisingly high dynamics of the DON/STV interaction, which is affected by the structure of the DNA linker system. The cell biological studies confirmed that cellular activation correlates with the stability of DON/STV interaction.

## 2 Results and Discussion

**Figure 1:**
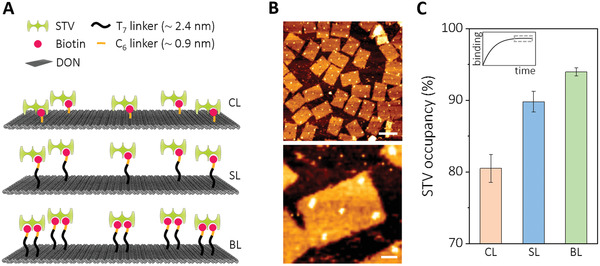
Schematic depiction of the biotinylated DON pegboards, dubbed as CL‐, SL‐ and BL‐DON for “conventional linker”, “single‐strand linker” and “bidentate linker”, respectively. Five binding positions for STV are present on each DON. Single‐stranded protruding staples at the bottom, used for immobilization on solid surfaces, are not shown for clarity. B) Representative AFM images of BL‐DON. Scale bars are 100 nm (top) and 20 nm (bottom image) C) STV occupancies obtained at equilibrium conditions (framed within the inset of a hypothetical binding curve) in the presence of excess STV. All measurements were done in triplicates (*N* > 250). Error bars correspond to the standard deviation obtained.

To study the influence of linker geometries on STV binding, we used a rectangular DON with dimensions of 70 × 100 nm^2^. The biotinylated staples were designed to carry monodentate biotin groups either linked by conventional C_6_ linkers to the 5’ end of the staples within the origami plane (in the following, dubbed as “conventional linker”, CL‐DON); or by C_6_ linkers tethered to single‐stranded dT_7_ oligomers protruding from the origami plane (“single‐stranded linker”, SL‐DON). In addition, a bidentate linker system was used in which two flexible dT_7_ strands were attached to adjacent staples that protrude from the origami plane and present two biotin residues in immediate proximity ("bidentate linker″, BL‐DON). A total of five binding sites for tetravalent STV were installed on the surface of the rectangular DON for each design (**Figure** **1**). For details on the origami and linker design and fabrication, see Figures S1 and S2 in the Supporting Information.

To determine the binding efficiency of STV to the different DON structures under equilibrium conditions, the STV occupancies were first characterized by conventional AFM imaging. To this end, 150 pmol of origami were incubated with 25 nmol of STV, corresponding to 33 molar equivalents of STV per biotin binding site, for 30 min in a volume of 50 µL TE‐Mg buffer. Aliquots of these solutions were then incubated on freshly cleaved mica for 3 min and analyzed by AFM (Figure 1B,C). Note that STV was present in excess during scanning. It was found that typical occupation densities of about 80 ± 2.3% could be reached with the CL system, whereas the addition of the flexible dT_7_ linker in the SL system led to substantial improvement to about 90 ± 1.5%. The use of the bidentate linker (BL) resulted in occupancy densities of about 94 ± 0.6% with only very small statistical variations. Conventional AFM scanning is known to have negligible negative effects on the binding efficiency of STV to DON structures.^[^ ^8^ ^]^ Therefore, the yields obtained under equilibrium conditions (Figure 1C) clearly reflect different binding affinities for the various constructs.

**Figure 2:**
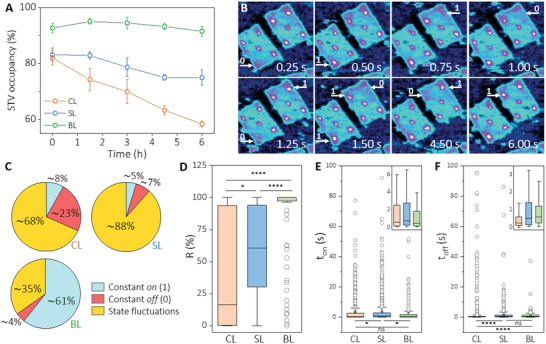
A) STV occupancies of the three DON measured by conventional AFM after repeated washing over 6 h time period. Measurements were done in triplicates. B) Representative HS‐AFM frames were obtained from the analysis of CL‐DON at different time points. Binding fluctuations at specific STV binding positions are marked by arrows, where a change of state occurs in the following frame. C) Proportion of STV binding positions that have either constant binding states (on/off, in blue/red respectively) or fluctuations (yellow) in binding states. For details, see text and Figure S3, Supporting Information. D) Calculated total residence, *R*, for the various DON systems determined for individual STV‐binding positions (*n* = 158 for CL, 129 for SL, 112 for BL). E) Duration of *t*_on_ and (F) *t*_off_ intervals observed for the same STV‐binding sites showing state fluctuations. Boxplots in D), E) and F) show median, 25^th^ and 75^th^ percentiles. Outliers are represented in circles. Data were analyzed using the non‐parametric Kruskal‐Wallis 1‐way ANOVA test on ranks and a Dunn‐Bonferroni test for pairwise comparisons; *P < 0.05 and ****P < 0.001 for comparisons between CL, SL, and BL‐DON. Exact values of means, interquartile ranges and *P* values are shown in Table S4, Supporting Information. Insets in D) and F) show a zoomed in view of the boxes and whiskers. Histograms for *t*_on_ and *t*_off_ values and a brief discussion on the outlier determination are shown in Figure S4, Supporting Information.

Considering that in typical cell experiments a DON‐decorated solid surface is often subjected to multiple washing steps, we wanted to investigate the effect of repeated washing on the STV occupancies. For this purpose, mica discs were glued to Petri dishes. DON were allowed to bind STV and incubated on the freshly cleaved mica as described above. Subsequently, the DON were washed with TE‐Mg buffer and 2 ml of fresh buffer was added. AFM measurements of these samples were performed every 90 min for a total of 6 h and after each measurement the buffer was replaced with fresh buffer. **Figure** **2**A shows the time‐dependent STV occupancies for the different DON constructs. While BL‐DON showed a negligible decrease from 93% to 91%, the monodentate SL and CL‐DON showed a progressive decrease in STV occupancy over time, which was smaller for SL (from 83% to 75%) than for CL (from 82% to 58%). These results, which suggest higher dissociation rates for the CL‐ and SL‐DON, also allow us to discard competition with the STV present in the solution and the STV bound to the DON.

Since these results suggested a higher dissociation rate for the CL‐ and SL‐DON compared to the BL‐DON, we investigated the binding dynamics between the STV and the biotinylated DON with the highest possible spatiotemporal resolution. To this end, we used, for the first time, a novel HS‐AFM instrument, which allows recording times as short as 50 ms per frame (corresponding to a scan rate of 20 frames per second) due to high line rates of 2 kHz. With these specifications, it is superior to the HS‐AFM methods previously used for DON characterization^[^ ^6^, ^9^ ^]^ (see also Table S3, Supporting Information). Specifically, the dynamics of STV binding of the different DON constructs were investigated by adsorbing DON onto freshly cleaved mica, then overlaying it with STV (200 nM in 1 mL TE‐Mg buffer), and then analyzing it using a NanoRacer HS‐AFM (JPK BioAFM, Bruker, Germany). All frames were analyzed separately, yielding time‐resolved data for a total of about 400 binding positions indicating the presence (classified as “1”) or absence (“0”) of STV on the DON. Surprisingly, we found that a substantial proportion of the STV positions in all DON systems showed binding fluctuations during the measurement period of about 105 s. Figure 2B shows representative frames exhibiting these intermittent binding events for the CL‐DON.

To better understand the fluctuating behavior, STV binding events were classified according to their duration and frequency (Figure 2C). We distinguished between binding events that show no fluctuation, those being divided into “constant on” (blue) and “constant off” (red), and binding events showing state fluctuations (yellow). Representative examples of each group and all exact values underlying the diagrams in Figure 2C are shown in Figure S3, Supporting Information. This classification showed that in the monodentate systems the constant on (blue) states never exceed 10% (8.2% CL and 4.6% SL), while in the BL system this state accounts for about 61%. Constant off (red) states were highest for CL (23%), followed by SL (7%) and BL (4%). Thus, these data support the expected stability of the linker systems derived from the conventional occupancy study (Figure 1C). Furthermore, the constant off values portions of SL and BL could be explained by missing or unlabeled staples. Hence, the high CL value suggests a reduced binding ability of this linker configuration, possibly due to steric hindrance which may affect the kinetic on‐rate of STV binding. Most striking, however, was the surprisingly high dynamics of the SL system, which led to the occurrence of 88% fluctuating sites, compared to 68% and 35% for the CL and BL systems, respectively. We note that the excess of STV contributes to the frequency of rebinding events. In the hypothetical case that similar measurements were done under continuous flow or washing conditions, where dissociated STV molecules would be washed away, no rebinding would occur.

A detailed analysis of the duration of binding between the STV and the DON structures was performed for all binding positions showing state fluctuations (yellow sections in Figure 2D). We calculated the “total residence” (*R*) for all positions to quantify the total time that a single STV molecule was bound at a given position relative to the total measurement time (*t*_m_). Results are given as percentages (*R* = ∑*t*_on_ × *t*_m_^–1^ × 100). The results, shown as a boxplot diagram in Figure 2D, clearly show that the three constructs exhibit a reduction in *R* variances that correlates with the differences in stability observed above. Specifically, while *R* is broadly distributed for CL, this variance is significantly lower for the SL and for the BL‐DON system. Of note, the data points classified as outliers in the BL system, may have lost one of the biotinylated staples for bivalent binding, thus showing the same behavior as in the monovalent SL system.

To further elucidate the dynamics of binding/unbinding, we analyzed the individual *t*_on_ (period of uninterrupted association, classified as “1”) and *t*_off_ (period of uninterrupted dissociation, classified as “0”) intervals during the total analysis time (for exact data and a graphical representation of state fluctuations, see Figure S3, Supporting Information). Start and end intervals were not included in this analysis because their exact duration could not be determined. Hence *t*_on_ and *t*_off_ reflect only the quantifiable dwell times within the total measurement time. As expected from the data in Figure 2C, the SL DON showed the highest number of fluctuations per STV site, followed by CL and BL (Figure S3, Supporting Information). Analysis of *t*_on_ values (Figure 2E) revealed only very small differences between CL and SL with only low statistical significance. However, a higher statistical significance was indicated for *t*_off_ intervals between SL and CL (Figure 2F). We hypothesize that longer *t*_off_ intervals may reflect a lower probability of stochastic collision between the flexibly arranged biotinylated staples and STV molecules in solution, due to the higher entropy of this single‐stranded linker. Indeed, logarithmic plots of the probability density functions of the *t*_on_ data for CL and SL suggest the presence of two types of on‐states, showing average *t*_on_ times of 0.06 s and 0.6 s (Figure S3E, Supporting Information). While in the CL system the short‐ and long‐lived states are almost equally populated, in SL this ratio shifts significantly in favor of the long‐lived states. Overall, these results consistently suggest that the stronger binding of SL compared to CL is associated with higher turnover dynamics. Considering the evidence obtained from Figure 2C for a lowered kinetic on‐rate of the CL system, these data suggest that its increased entropy affects the kinetic on‐rate more than the off‐rate.

Note that in the processing of the binding data, by restricting to transient binding events, about 80% of the binding times observed for the BL‐DON were removed (Figure S2D, Supporting Information) because they did not fluctuate within the total measurement time. Hence, the analysis of the *t*_on_ and *t*_off_ is not considered highly relevant and representative for BL binding. Nevertheless, as shown in Figure S2C, Supporting Information, in BL‐DON most STV molecules that exhibit state fluctuations are bound for long periods of time and rarely dissociate. Therefore, due to the longer residence times, stronger bivalent binding correlates with lower fluctuation dynamics (see also Figure S3, Supporting Information).

Furthermore, data analysis of STV binding events also revealed non‐significant differences in duration and binding frequency depending on the position of binding sites on the DON (Figure S5, Supporting Information). While continuous fluctuations were measured for all DON systems, we also observed a decrease in the overall STV occupancy over the measurement period (Figure S6, Supporting Information). This occurred particularly in the CL and SL systems, where the mean STV occupancy decreased by about 40% over the course of the experiment despite the possibility of STV rebinding. However, on the BL construct, which exhibits the strongest interaction, the majority of STV bonds remain intact over time despite the forces exerted, so that the mean occupancy shows a decrease of ≤10% over the total measurement time. This result clearly reflects the mechanical stress from the probe tip during high‐speed scanning, which has previously been shown to negatively affect the monovalent binding between STV and biotinylated DON structures.^[^ ^6^ ^]^ For a direct comparison of the tip effects observed here and previously,^[^ ^6^ ^]^ the obtained loss curves were analyzed by applying an exponential decay fit to quantify the tip‐induced dissociation rate constants (*k*_off,tip_). Data analysis showed that this detrimental effect of HS‐AFM resulted in similarly large STV losses observed in the previous study that was conducted with an approximately 100 times slower scanning rate (0.1 frames per second (fps),^[^ ^6^ ^]^ instead of 20 fps used in here (see also Figure S4 and Table S3, Supporting Information). See Figure S6, Supporting Information, for exact values and graphical representations. We note that due to tip‐induced dissociation, one would expect that an increase in measurement time would lead to lower amount of constant on states and higher amount of fluctuations in Figure 2C.

**Figure 3:**
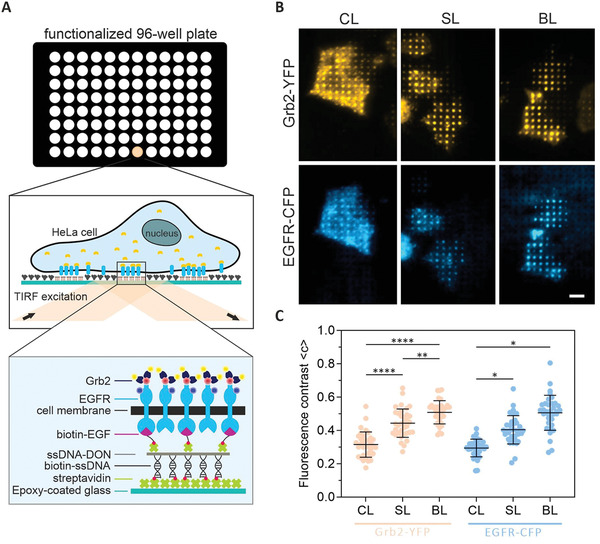
A) Schematic depiction of the multi‐well plate‐based platform used for the subcellular micropatterning experiments on DON functionalized surfaces. B) Representative TIRF microscopy images of HeLa cells transiently co‐expressing EGFR‐CFP and Grb2‐YFP and grown on the different DON‐micropatterned substrates. Scale bar: 5 µm. C) Quantitation of EGFR‐CFP and Grb2‐YFP fluorescence contrast. Error bars are based on the mean ± SD of more than 30 cells measured from at least two independent experiments. *P < 0.05, **P < 0.01 and ****P < 0.001 for comparisons of CL, SL and BL DONs using one‐way ANOVA followed by Holm‐Sidak's multiple comparisons test.

To investigate whether the above described differences of the various linker systems have an impact on the biological function of the constructs, we studied the activation of the epidermal growth factor receptor (EGFR) using living HeLa cells that were allowed to adhere on surface‐bound DONs decorated with biotinylated epidermal growth factor (bEGF). For this purpose, we used our recently developed multi‐well plate‐based platform for subcellular micropatterning experiments.^[^ ^1^, ^10^ ^]^ In this method, EGFR molecules are arranged in the living cell membrane by interaction with micro/nano patterns of EGF ligand on solid substrates. By fluorescence microscopic analysis of the downstream recruitment of the adapter protein Grb2, as a parameter of EGFR activation, statements on the biological efficacy of the bEGF‐STV‐DON structures can then be obtained (**Figure** **3**). To this end, the three different STV‐DON constructs were incubated with bEGF and then immobilized via DNA hybridization onto micropatterned solid substrates (Figures 3A and S7, Supporting Information). In brief, epoxysilane‐coated glass slides were initially protein patterned by means of large‐area microcontact printing using an elastomeric stamp that contained a continuous 1 µm grid pattern to facilitate transfer of a micron‐scale BSA grid (for surface passivation) onto the activated glass substrate. Next, STV was immobilized in the resulting unblocked 1 µm patterns, and biotinylated single‐stranded (ss‐) DNA capture‐oligonucleotides were bound to the STV. The different DONs, equipped with ssDNA linker strands protruding from the origami plane, were than bound on the surface via specific Watson‐Crick hybridization. See the Experimental Section/Methods for a detailed description on the washing steps. Once the surface was fully functionalized with DON‐STV‐bEGF constructs, HeLa cells, expressing the respective fluorescent fusion proteins, were incubated on the surface for at least 3 hours and analyzed by total internal reflection fluorescence (TIRF) microscopy.

Figure 3B shows representative TIRF microscopy images of HeLa cells transiently expressing EGFR‐CFP and Grb2‐YFP that were grown on the DON‐micropatterned substrates. In line with our previous study, we detected a high degree of specific EGFR and Grb2 co‐patterning in cells grown on EGF‐decorated DONs with the CL‐DON system (<cEGFR> = 0.29 ± 0.03; <cGrb2> = 0.32 ± 0.01). Grb2 is known to directly bind to phosphorylated tyrosine‐containing peptides on receptors (such as EGFR) via its SH2 domain, which results in the activation of downstream kinases.^[^ ^11^ ^]^ Hence, the amount of Grb2 corecruited to the EGFR can serve as a parameter for EGFR activation subject to the different DON‐patterned surfaces. In concordance with the results obtained, when cells were grown on SL‐DONs, a significantly higher enrichment of the EGFR (<c> = 0.40 ± 0.04; *P* <0.05) as well as Grb2 copatterning (<c> = 0.44 ± 0.01; *P* < 0.001) was measured, as compared to the CL system. Implementation of BL‐DONs even led to further EGFR patterning (<c> = 0.51 ± 0.06) and activation, resulting in an enhanced Grb2 corecruitment (<c> = 0.51 ± 0.01; *P* < 0.01 as compared to SL). The only minor differences in EGFR recruitment observed between BL and SL could be due to several reasons. First, Grb2 copatterning but not enrichment for the receptor is occasionally observed, which could be due to different expression levels of EGFR and Grb2. On the other hand, the error bars show that EGFR contrast levels are not as stable as Grb2 levels, resulting in non‐significant conditions, but still clearly demonstrating increased EGFR patterning for the BL system.

## 3 Conclusion

In summary, our study shows that engineering of the linker system has a substantial impact on the dynamics of ligand presentation on DON and, consequently, on biological functionality. Although it has recently been reported that increasing lengths of the spacer improve the binding efficiency of antibody‐DNA conjugates and thus of DON to the cellular receptor,^[^ ^12^ ^]^ here we demonstrated for the first time that linker engineering via STV‐biotin bridges has a direct effect on ligand‐mediated cellular activation. The nature of the linker influences not only changes in global functionalization efficiency, but also the lability and consequent loss of ligands in the course of cell biological experiments with successive washing steps. Therefore, to ensure stable DON‐STV binding over a longer period of time, bidentate linkers should preferably be used. Stability and dynamics of DON‐Ligand systems are also likely to affect the number and duration of interactions with cells. Our work suggests that the design of ligand‐functionalized DON can be used to modulate not only steric accessibility but also the transient dynamics of the interaction between the artificial and biological systems. Thus, we believe that this work represents an important contribution to the further standardization of DNA nanostructures for the study of biological processes.

## 4 Experimental Section

### Design of the DNA Origami Nanostructures

The general design of the rectangular DNA origami nanostructures (DON) used in this work was adapted from Rothemund's design.^[^ ^13^ ^]^ The DONs were assembled using the single stranded scaffold p7560 (Tilibit Nanosystems) and 240 staple strand oligonucleotides (Sigma Aldrich). These DONs were twist‐corrected by deleting one base from every fourth column of staples. B‐DNA has a helical twist of 10.4 base pairs/turn (bp/turn). Without twist correction, the helices on the DNA origami would have a helical twist of 10.67 bp per turn, which induces a significant global twist of the DON. By deleting one base from every fourth column of staples, a helical twist of 10.53 bp per turn was achieved, which in turn translates into a flatter DON.^[^ ^14^ ^]^ Five positions were chosen on the DON's upper side for the installment of biotinylated staples for the subsequent streptavidin (STV) binding (these staples were colored in orange in the design schematics, see Figure S1, Supporting Information). The main text contains a detailed explanation of the design of the biotinylated staples. Additionally, nine positions were chosen on the lower side of the DON for the incorporation of single‐stranded binding tags. These protruding single‐stranded tags allow the immobilization of the DON on a solid surface via hybridization with surface‐bound capture oligonucleotides (these staples were coloured in red in the design schematics, see Figure S1, Supporting Information).^[^ ^1b^ ^]^ The selected staples were elongated 24 bases. In this work, the sequence for the binding tags called here “cTr1” was used. With “lower” and “upper side”, the authors refer to the position on the DON plane after binding on a surface. The sequences of unmodified staple strands are listed in Table S1, Supporting Information. All the modifications were realized by exchanging the respectively modified staples, as specified in Table S2, Supporting Information.

### Assembly, Purification, and Quantification of DNA Origami Nanostructures

DONs were assembled according Rothemund's procedure,^[^ ^13^ ^]^ using a 1:10 molar ratio between the scaffold strand p7560 and each of the staple strands. The assembly was conducted in TE‐Mg 7 buffer (20 mM Tris base, 1 mM EDTA, 7 mM MgCl_2_, pH 7.6 adjusted with HCl) in a total volume of 500 µL. The DON were assembled on a Thermocycler (Eppendorf Master cycler pro) by a step‐wise temperature decrease from 75 to 25 °C. After an initial denaturing step at 95 °C for 5 min, the temperature was decreased at −1 °C per cycle for 50 cycles and each step was held for 10 s. After annealing, excess staple strands were removed by PEG precipitation according to Dietz's procedure.^[^ ^15^ ^]^ The DON were precipitated by adding a 1:1 volume ratio of precipitating buffer (5 mM Tris base, 1 mM EDTA, 505 mM NaCl, 15% PEG‐8000), followed by centrifugation at 16 000 g for 30 min. The obtained pellet was resuspended in 50 µL TE‐Mg 6 buffer (20 mM Tris base, 1 mM EDTA, 6 mM MgCl_2_, pH 7.6). The concentration of the purified DONs was determined by a quantitative polymerase chain reaction (qPCR) as previously reported.^[^ ^1b^ ^]^

### Conventional AFM Measurement

For the conventional AFM characterization, 150 pmol DONs were incubated with 25 nmol of STV in a final volume of 50 µL for 30 min in TE‐Mg 12.5 buffer (20 mM Tris base, 1 mM EDTA, 12.5 mM MgCl_2_, pH 7.6). Then, 10 µL of this solution were added to freshly cleaved mica (Plano GmbH). After 3 min incubation, 50 µL of TE‐Mg 12.5 buffer were added. The samples were imaged with pyramidal tips (SNL‐10 tips, radius 2 nm, spring constant 0.35 N/m, Bruker) using a NanoWizard 3 atomic force microscope (JPK) under a force‐curve based imaging mode (QI) with a setpoint of about 100 – 200 pN. The obtained images were analyzed by using the JPK data processing software.

To assess whether fluctuations can also be seen by standard AFM measurement, a certain area on the mica previously incubated with CL‐DON was repeatedly measured for four times. In this case, TE‐Mg 12.5 buffer containing 200 nM STV was added to the mica surface decorated with DON. Due to the resolution (256 × 256 px^2^) and the line rate (1 Hz), every image took about 4 min to be scanned. Due to the low temporal resolution, it is not possible to observe fluctuations occurring in the millisecond/second range and reproduce the results obtained by HS‐AFM. However, sporadic fluctuations could be detected, as shown in Figure S9, Supporting Information.

### High‐Speed AFM Measurement and Statistical Analysis

For the high‐speed AFM characterization, 20 µL of 2.5 nM DONs were incubated on freshly cleaved mica for 5 min. Then 1 mL of STV 200 nM in TE‐Mg 12.5 was added and the DONs were imaged at a scan speed of 20 fps for about 105 s using a NanoRacer HS‐AFM (JPK BioAFM, Bruker, Germany) and USC‐F1.2‐k0.15 tips (radius <10 nm; spring constant 0.15 N m^−1^, NanoWorld). Individual frames were extracted from the HS‐AFM videos and the STV positions on all origami were analysed for either presence (1) or absence (0) of STV. A total of 399 binding positions (158 for CL, 129 for SL, and 112 for BL) were analyzed. A script was developed in MATLAB for the calculation of the duration of the individual time intervals of uninterrupted association (*t*_on_, classified as “1” during the analysis), dissociation (*t*_off_, classified as “0” during the analysis) and the binding frequency, defined as the number of binding events. The obtained t_on_ was used for the calculation of the “total residence” (*R*) to quantify the total time that a single STV molecule was bound at a given position relative to the total measurement time (*t*_m_). Results are given as percentages (*R* = ∑*t*_on_ × *t*_m_^–1^ × 100). Total residence (*R*), *t*_on_ and *t*_off_ values obtained for the three different DON were subjected to the nonparametric Kruskall–Wallis 1‐way ANOVA significance test to analyze the differences between the three DON systems. Dunn‐Bonferroni post hoc test was used for pairwise comparisons. *P* < 0.05 was considered as statistically significant. Exact values of means, interquartile ranges (IQR) and *P* values for pairwise comparisons are shown in Table S4, Supporting Information. Outliers were determined as all points lying outside the interval [*Q_1_* − 1.5 IQR; *Q_3_* + 1.5 IQR]. See Figure S4, Supporting Information, for a discussion on the outlier determination. Comparisons for the total residence, *R*, between inner and outer positions on the origami were done using a Mann–Whitney U‐test. *P* < 0.05 was considered as statistically significant. All analyses were carried out with the SPSS statistical package.^[^ ^16^ ^]^ Furthermore, the STV binding state data over time of all STV positions for a given DON system were averaged for the calculation of the mean STV occupancy (yield) over time (Figure S6, Supporting Information). These curves were analyzed by applying an exponential decay fit according to literature^[^ ^6^ ^]^ for the calculation of the tip‐induced dissociation rate constants (*k*_off,tip_). The equation was modified to account for the different STV occupancy yields at time 0 Equation 1.

(1) $$\text{yield}=\text{ySS}+(y0-y\text{SS})e^{-k_{\text{off,tip}}(t-t0)}$$

being *y*SS the STV occupancy at the stationary state and y0 the STV occupancy at time 0.

### Cell Culture and Transfection

All cell culture reagents were purchased from Biochrom. HeLa cells (ATCC) were cultured in RPMI medium supplemented with 10% fetal bovine serum (FBS) and 1% penicillin/streptomycin and grown at 37 °C in a humidified incubator with 5% CO2. For transient transfection, HeLa cells were sub‐cultured the day before and were then transfected with plasmids using jetOPTIMUS DNA transfection reagent (Polyplus transfection, Illkirch, France), according to the manufacturer's instructions. HeLa cells stably expressing Grb2‐YFP were described previously.^[^ ^17^ ^]^

### Microcontact printing

Microcontact printing was performed as described previously.^[^ ^1^, ^10^ ^]^ In short, a field of a large‐area PFPE elastomeric stamp (1 µm grid size), obtained by the EV‐Group (St. Florian am Inn), was cut out and washed by flushing with ethanol (100%) and distilled water. After drying with nitrogen, the stamp was incubated in 50 mL bovine serum albumin (BSA) solution (1 mg mL^−1^) for 30 min. This step was followed by washing the stamp again with phosphate‐buffered saline (PBS) and distilled water. After drying with nitrogen, the stamp was placed with homogeneous pressure onto the clean epoxy‐coated glass bottom of a 96‐well plate and incubated overnight at 4 °C. The next day, the stamp was stripped from the glass with forceps, and the glass bottom was bonded to a 96‐well plastic casting using adhesive tape (3M) and closed with an appropriate lid.

### Live Cell Micropatterning – DNA Origami Patterning

DNA micropatterns were assembled on microcontact printed substrates as follows: first, the reaction chamber (wells of the multi‐well plate) was incubated with 100 µL STV (50 µg mL^−1^) for 30 min at room temperature and then washed four times with TETBS‐150 (20 mM Tris base, 150 mM NaCl, 5 mM EDTA, and 0.05% Tween‐20 [v/v], pH 7.5), twice for 30 s each time and twice for 5 min each time. Next, 50 µL of biotin‐Tr1‐12 oligonucleotide (240 nM, 5′‐[Btn] TTTTTTTT ATG ATG ATG ATG‐3′) was added and incubated at room temperature for 30 min with continuous shaking. The chambers were washed three times with biotin‐TETBS (20 mM Tris base, 150 mM NaCl, 5 mM EDTA, 0.05% Tween‐20 [v/v], and 800 µM D‐biotin), twice for 30 s each time, and once for 20 min at room temperature, which was followed by two TE‐750 (10 mM Tris base, 1 mM EDTA and 750 mM NaCl) washes for 1 min each. DON preparation was performed by mixing 100 fmol biotinylated DONs with 1.67 µL STV (10 µM) and with PBS‐/‐ (Thermo Fisher Scientific) added to a total volume of 10 µL and incubating the mixture for 30 min while shaking at room temperature. Subsequently, 5 µL of biotin‐EGF (6 µM) was added and incubated for 45 min while shaking at room temperature. Next, 20 µL of biotin‐TETBS‐150 was added, and the solution was again incubated for 15 min at room temperature while shaking. Finally, 50 µL TE‐750 was added to the mixture. For DON hybridization, 90 µL of the DON mixture was incubated on the oligonucleotide‐functionalized surfaces for 2 h at room temperature. The chambers were washed 4 times with TE‐750, twice for 30 s and twice for 5 min, followed by 2 washes with PBS‐/‐for 1 min each time. Note that the exact number of wash steps in this protocol has been optimized and is critical to ensure the reproducibility of the experiments. Changing the number of wash steps can lead to significantly lower reproducibility, presumably due to non‐specifically bound DON on the glass surface, so such variations do not allow conclusions to be drawn about the STV occupation density on the DON. Subsequently, the cells were seeded and incubated at 37 °C for approximately 3 h to ensure a homogeneous cell membrane/substrate interface, which was followed by live cell microscopy analysis via TIRF microscopy.

### Live Cell Micropatterning – Live Cell TIRF Microscopy

The detection system was set up on an epi‐fluorescence microscope (Nikon Eclipse Ti2). A multi‐laser engine (Toptica Photonics) was used for selective fluorescence excitation of CFP and YFP, at 405 and 516 nm, respectively. The samples were illuminated in total internal reflection (TIR) configuration (Nikon Ti‐LAPP) using a 60x oil immersion objective (NA = 1.49, APON 60XO TIRF). After appropriate filtering using standard filter sets, the fluorescence was imaged onto a sCMOS camera (Zyla 4.2). The samples were mounted on an *x*‐*y*‐stage (CMR‐STG‐MHIX2‐motorized table), and scanning of the larger areas was supported by a laser‐guided automated Perfect Focus System (Nikon PFS). Figure S8, Supporting Information, shows the cell boundaries to help the reader identify the cells.

### Live Cell Micropatterning – Contrast Quantitation and Statistical Analysis

Contrast analysis was performed as described previously.^[^ ^18^ ^]^ Initial imaging recording was supported by the Nikon NIS Elements software. Images were exported as TIFF frames and fluorescence contrast analysis was performed using the Spotty framework.^[^ ^19^ ^]^ The fluorescence contrast <c> was calculated as <c> = (F^+^‐ F^–^)/(F^+^‐ F_bg_), where F^+^ denotes the intensity of the inner pixels of the pattern. F^–^ shows the intensity of the surrounding pixels of the micropattern, and F_bg_ shows the intensity of the global background. Data are expressed as the means ± SD. For significance testing, more than two groups were compared with one‐way ANOVA, which was followed by Holm‐Sidak's multiple comparisons test.

### Statistical Analysis

Data were processed using SPSS (Chicago, IL, USA) or Graphpad Prism software (San Diego, CA, USA) and graphed using Origin (Northampton, MA, USA) or Graphpad Prism. Data are represented as boxplots showing median, 25^th^ and 75^th^ percentiles, or as means ± SD. Statistical analyses were carried out by performing Kruskal‐Wallis one‐way ANOVA followed by post hoc Dunn‐Bonferroni test, or one‐way ANOVA followed by post hoc Holm‐Sidak's test, as appropriate. Statistical significances (indicated by asterisks) of *P* < 0.05 (*), *P* < 0.01 (**), and *P* < 0.001 (****) were reported for differences between groups. Sample sizes are indicated along the description of the graphs within the corresponding figure captions. For specific analyses see sections High‐Speed AFM measurement and statistical analysis and Live cell micropatterning – Contrast quantitation and statistical analysis in the Experimental section/Methods. We also note that by using a large‐scale imaging approach in combination with the large‐scale micropatterning assay, The authors recently demonstrated that 90%–100% of cells exhibited significant bait‐prey copatterning when cells were presented with EGF‐decorated DONs. In contrast, the authors found less than 1% of the cell population exhibited nonspecific bait‐prey copatterning without presenting an appropriate anti‐bait protein (e.g., surfaces carrying only single‐stranded oligonucleotides or hybridized DONs without specific ligand).^[^ ^1h^ ^]^ Similarly, It had been previously shown that slight variation in DON coating on the plate surface had little effect on cellular activation.^[^ ^1b^ ^]^ For these reasons, and because in the present work the individual experiments with the different DON constructs were performed on the same surface (a microtiter plate‐see Figure S7, Supporting Information), artifacts due to different densities of occupancy of the glass surface with DON are highly unlikely.

## Conflict of Interest

The authors declare no conflict of interest.

## Supporting information

Supporting Information

Supplemental Movie 1

Supplemental Movie 2

Supplemental Movie 3

## Data Availability

The data that support the findings of this study are available from the corresponding author upon reasonable request.
