# Supplementary material for: Linker Engineering of Ligand‐Decorated DNA Origami Nanostructures Affects Biological Activity
Source: Small. 2022 Aug 7;18(35):2202704. doi: 10.1002/smll.202202704 (PMC11475364; doi:10.1002/smll.202202704)
Supplement: Supplementary file 1 — Supporting Information [file SMLL-18-2202704-s002.pdf]

NANO MICRO small

Supporting Information

for *Small,* DOI: 10.1002/smll.202202704

Linker Engineering of Ligand-Decorated DNA Origami Nanostructures Affects Biological Activity

Carmen M. Domínguez, Miguel García-Chamé, Ulrike Müller, Andreas Kraus, Klavdiya Gordiyenko, Ahmad Itani, Heiko Haschke, Peter Lanzerstorfer, Kersten S. Rabe, and Christof M. Niemeyer*

Supporting Information

Linker engineering of ligand-decorated DNA origami nanostructures affects biological activity

Carmen M. Domínguez, Miguel García-Chamé, Ulrike Müller, Andreas Kraus, Klavdiya Gordiyenko, Ahmad Itani, Heiko Haschke, Peter Lanzerstorfer, Kersten S. Rabe, and Christof M. Niemeyer.*

Table of Contents

	Page
1. Supporting Figures	2
2. Supporting Movies	12
3. Supporting Tables	13
4. References	22
5. Author Contributions	22

1. Supporting Figures

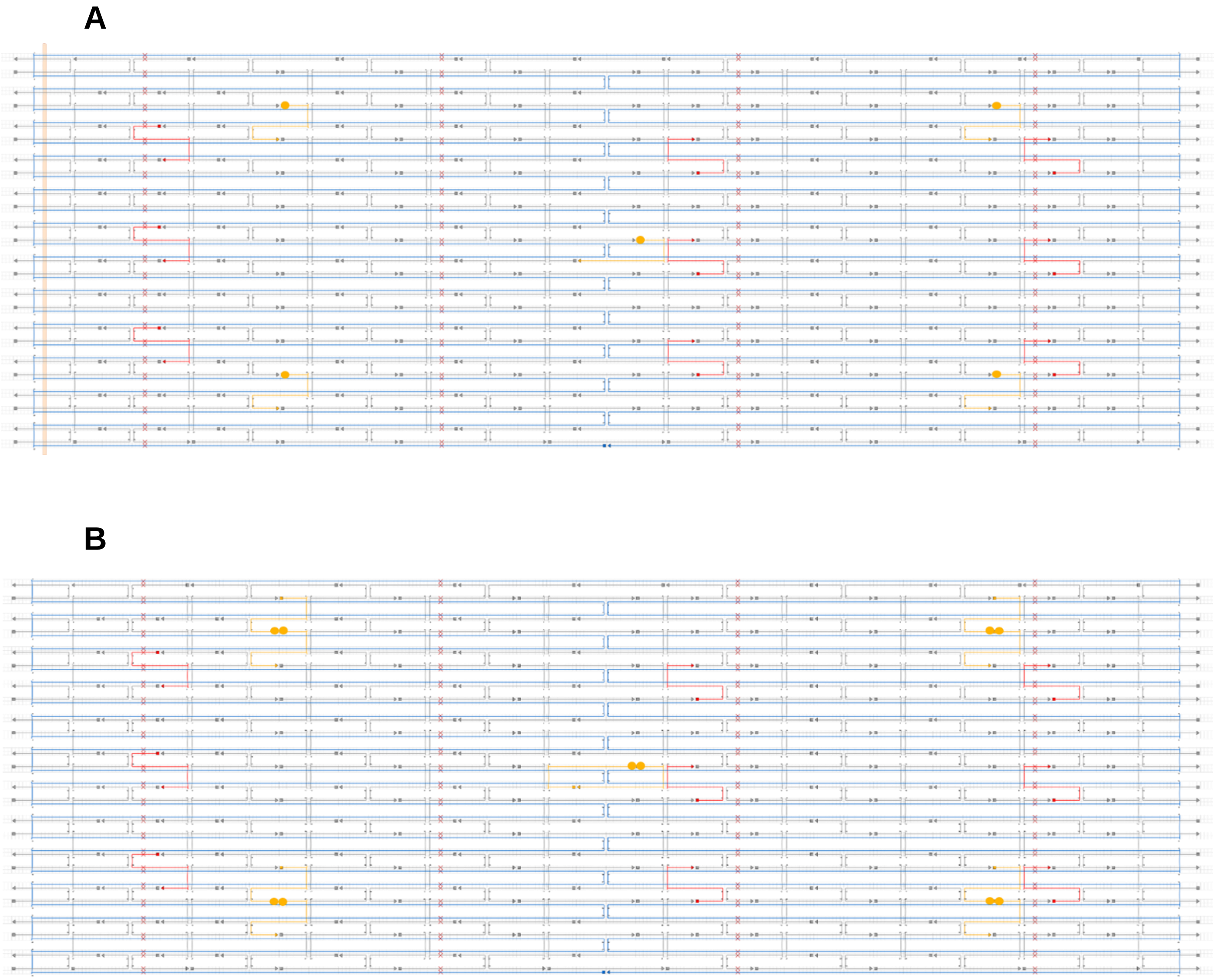

Figure S1. Design of DNA origami nanostructures (DON) as illustrated using the caDNAno software.[1] A) Design of the CL ("conventional linker") and SL ("single-strand linker") DON. Orange circles depict the 5' end for the protruding biotins. B) Design of the BL ("bidentate linker") system. Orange circles depict the 3' and 5' ends for the protruding biotins in close proximity. Red staples in A) and B) depict 3'-protruding staple strands on the lower side for hybridization on the surface.

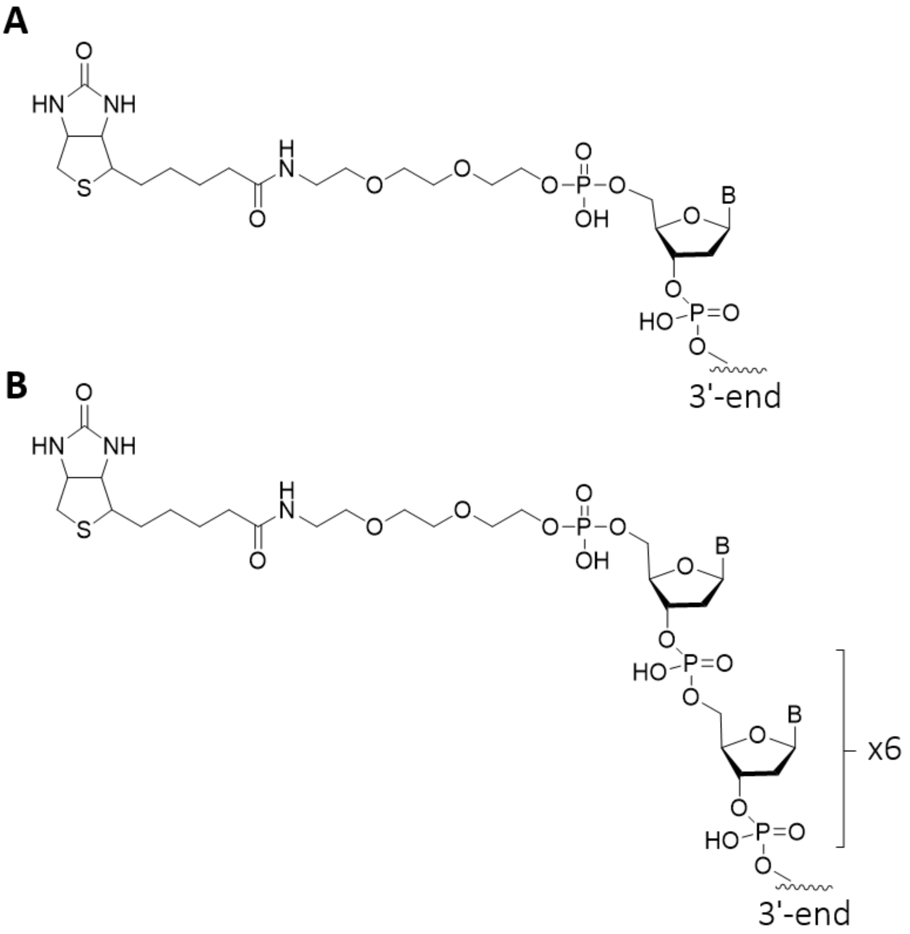

Figure S2. A) Representation of the chemical structure of the CL linker showing the biotin coupled to the 5' end of the staple within the DON via a C_6 conventional linker. B) Representation of the chemical structure of the SL linker showing the biotin coupled to the 5' end of the staple within the DON via a C_6 conventional linker plus a T7 tail. Note that the nucleobase is represented by the letter B and the chemical structure of thymine is not drawn.

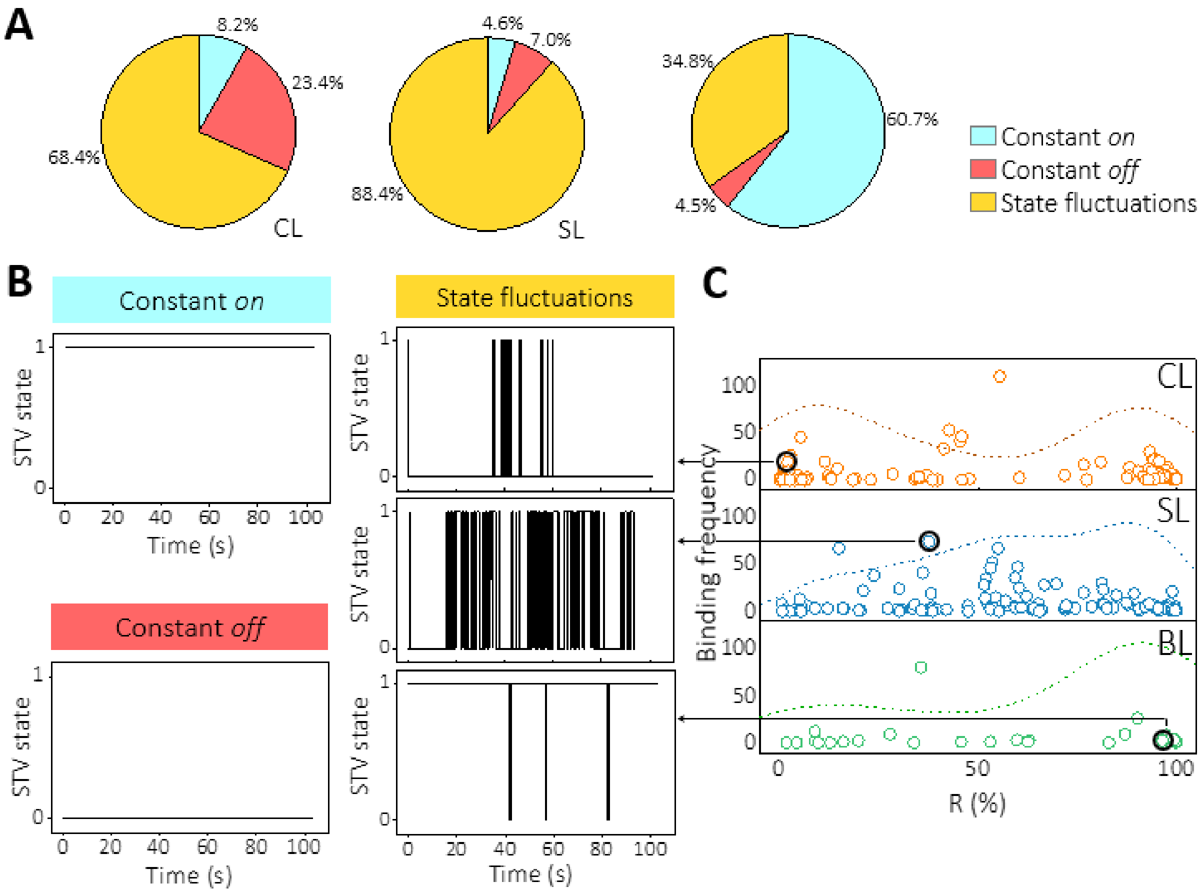

D

State fluctuations	CL	SL	BL
# of On Events	774	1128	167
STV sites	108	114	39
# of On Events /STV site	7.2	9.9	4.3

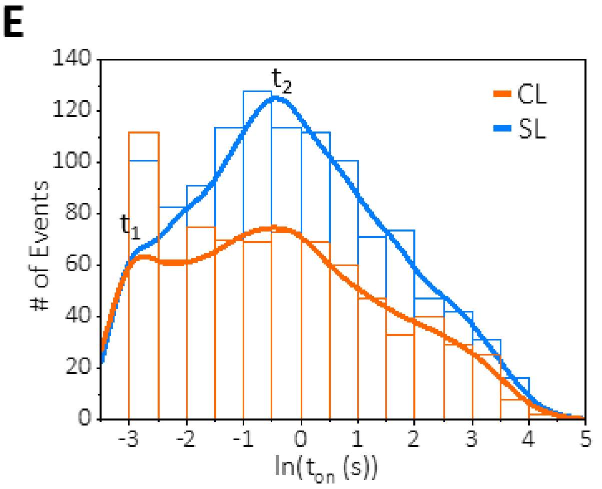

Figure S3. A) Proportion of STV binding positions that have either constant binding states (on/off, in blue/red respectively) or fluctuations (yellow) in binding states. B) Schematic illustration of the different classes of STV binding events, showing constant on (blue), constant off (red) and three different examples of fluctuating states (yellow). (C) Correlation between binding frequency and residence time R for the group of binding sites that exhibited state fluctuations (yellow sections in Figure S2A). Kernel density estimation (KDE)[2] curve fits obtained for the different R distributions are overlaid as dashed lines. The amplitude of the peaks is given by the relative frequency. Note that the Y-axis that correspond to these amplitude

values are the right Y-axis and values are not shown. D) Count of the individual ton intervals observed for the STV-binding sites that exhibit state fluctuations. Start and end intervals were excluded because their exact duration could not be determined. E) Logarithmic histogram of the ton data for the CL and SL-DON. KDE fits are overlaid.

Discussion

Figure S2C shows that CL has two main populations with low and high R, both with relatively low binding frequency. In contrast, the distribution of R values in SL DON is spread over a wide frequency range and shows an accumulation at higher R values. In BL-DON, fluctuations accumulate at low frequencies and high R values. The circled examples show the low frequency/low R binding that occurs on the CL-DON (top) as well as the high frequency/intermediate R binding that occurs on the SL-DON (middle) and the low frequency/high R binding that occurs predominantly on the BL-DON (bottom). The presence of populations with different lifetimes is even more evident in Figure S2E. Histograms with logarithmic binning are commonly used in the fields of protein folding and ion channels [3] since they allow for systematic identification of different characteristic timescales. The probability density functions suggest the presence of two types of on-states with average ton times of 0.06 s (t_1) and 0.6 s (t_2).

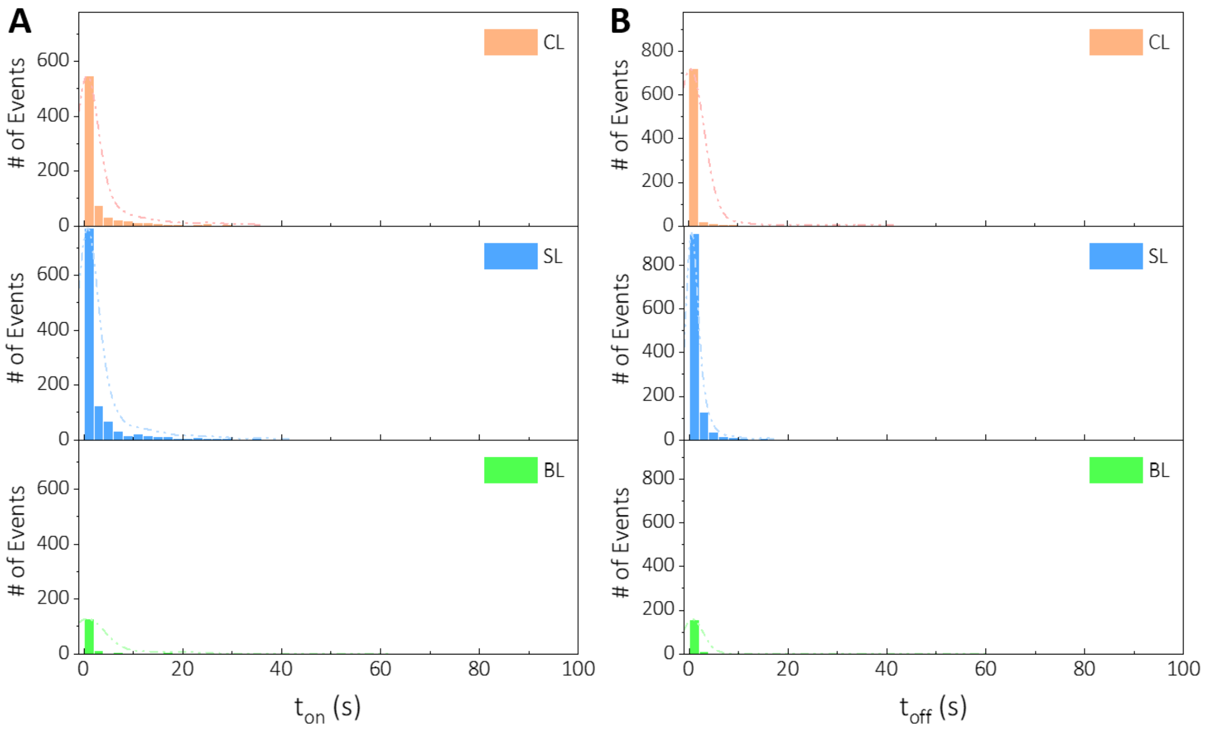

Figure S4. Histograms obtained for the t_{on} (A) and t_{off} (B) values shown in boxplots in Figure 2E and F. Kernel density estimation (KDE)[2] curve fits obtained for the different distributions are overlaid as dashed lines. Note that the distributions of t_{on} and t_{off} values are not normal, but right skewed. Hence, when the classical Tukey's formula is used to define outliers as all data points lying outside the interval [Q_1 – 1.5 IQR; Q_3 + 1.5 IQR], the probability of a data point to lie beyond the whiskers is much higher than in a normal distribution (see the amount of outliers shown in Figure 2E and F).[4] By doing a more advanced mathematical adjustment for skewed distributions, one could obtain longer whiskers and a great proportion of the outliers in a Tukey analysis would not be classified as outliers. This method is explained in [4-5].

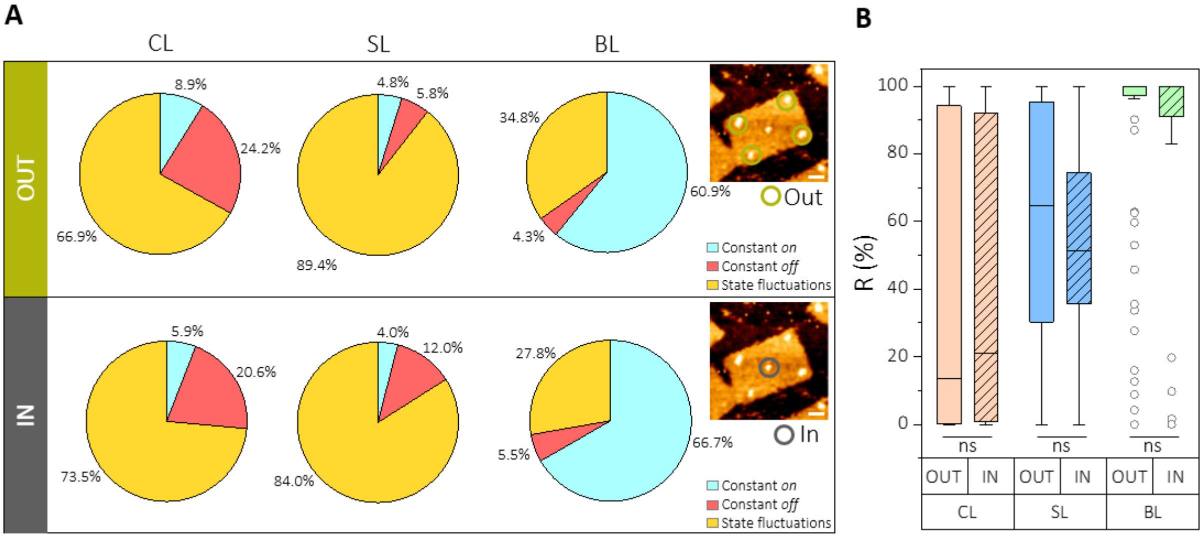

Figure S5. Site-dependent duration and frequency of the STV binding events. A) Proportion of STV binding positions that show constant binding states (constant on or constant off, in blue or red, respectively) or fluctuations (yellow), determined for outer (top, green) or inner (bottom, grey) positions on the three different DON systems. B) R determined for the outer and inner STV positions for the three different DON systems. "Out" and "in" groups were compared by the nonparametric Mann–Whitney U-test, revealing no significant differences.

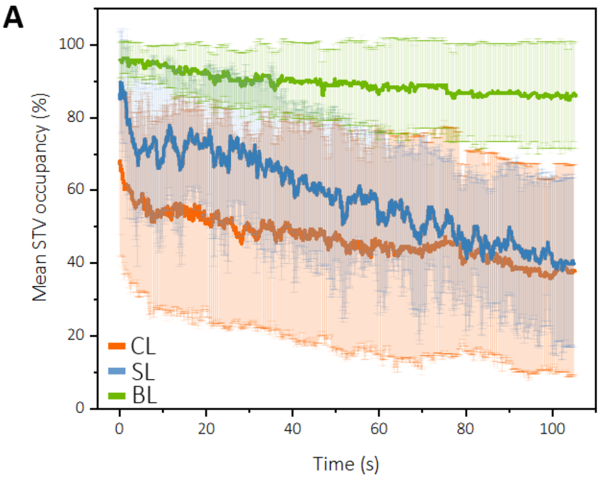

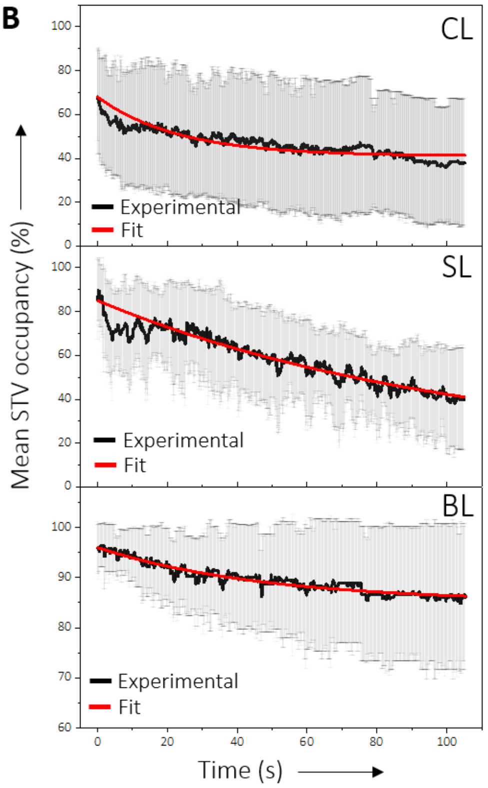

C

Linker	Line rate (Hz)	$k_{off,tip}$ (s^{-1})	Reference
Monodentate (~2.26 nm)	30 - 70	~0.005 – 0.07	[6]
Monodentate (CL, ~0.9 nm)	2000	0.04	This paper
Monodentate (SL, ~3.3 nm)	2000	0.01	This paper
Bidentate (BL, ~3.3 nm)	2000	0.02	This paper

Figure S6. A) Mean STV occupancy observed for the three DON designs during the full HS-AFM measurement time (n = 399 STV positions, 158 for CL, 129 for SL, 112 for BL). The shaded area corresponds to the SD obtained for each time point (50 ms). Note that the tip effects during high-speed scanning clearly affect weaker binding systems (CL and SL), where STV occupancy decreased by about 40% over the course of the experiment despite the possibility of STV rebinding. However, on the BL construct, which exhibits the strongest interaction, the majority of STV bonds remain intact over time despite the forces exerted, so that the mean occupancy shows a decrease of ≤10% over the total measurement time. This result is a clear indication of the mechanical stress generated by the scanned tip at high scan rates, which has previously been shown to negatively affect the monovalent binding between STV and biotinylated DON structures.[6] B) For a direct comparison of the tip effects observed here and previously,[6] we performed an exponential decay fit to calculate the tip-induced dissociation rate constant ($k_{off,tip}$). The results listed in the table (C) remarkably show that similar values of $k_{off,tip}$ from 0.005 to 0.07 s^{-1} are obtained, although in the previous study scanning line rates of 30 - 70 Hz were reported while in the present work much higher scanning line rates of 2 kHz were used. This indicates that the present instrumentation allows an almost 30-fold increase in the sampling rate without relevant adverse effects on the stability of STV-DON binding.

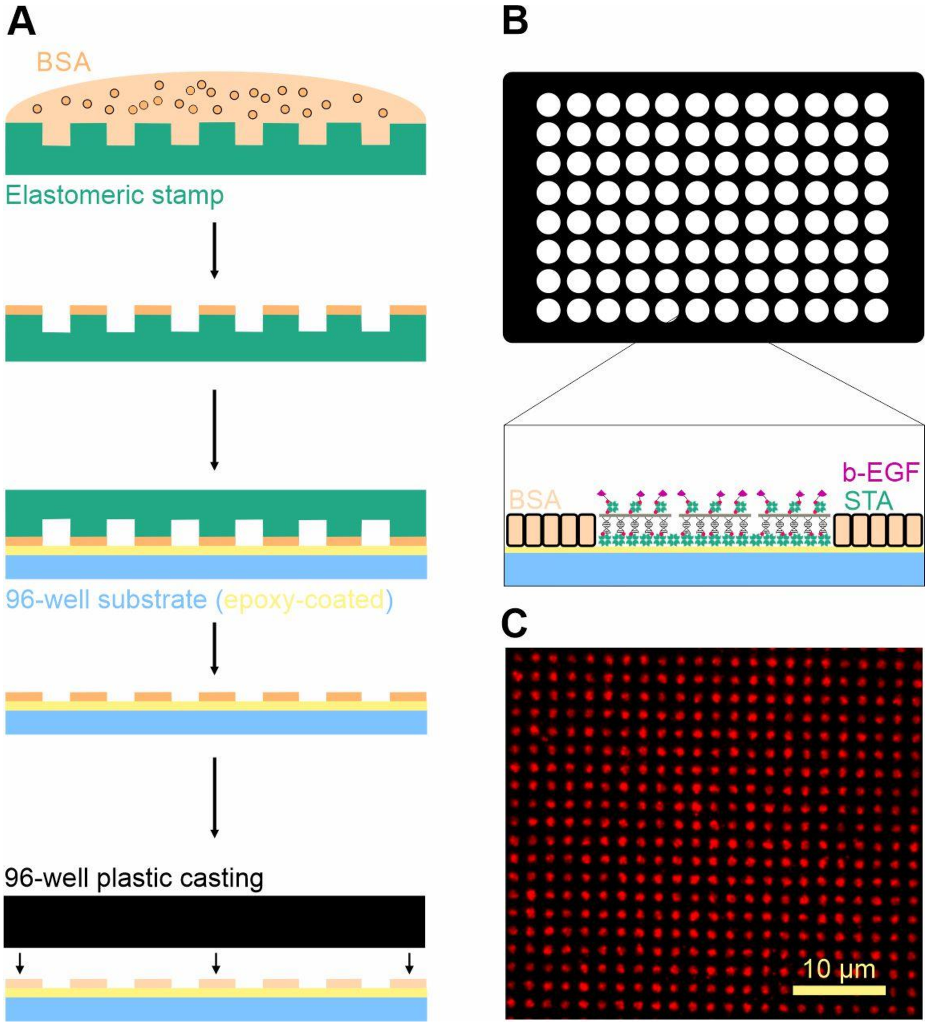

Figure S7. Cross-section sketches of the contact printing procedure for large-area protein micropatterns. (A) An elastomeric stamp is incubated with BSA followed by a washing step. Subsequently, the stamp is pressed onto an epoxy-coated glass substrate resulting in a transfer of the micron-scaled BSA grid. Finally, the microstructured glass is bonded with a multi-well plastic casting. (B) Ready-to-use multi-well plate for DON-functionalization and live-cell experiments. (C) Representative TIRF-microscopy image of 1 µm BSA grids consisting of hybridized Cy5-labelled DON.

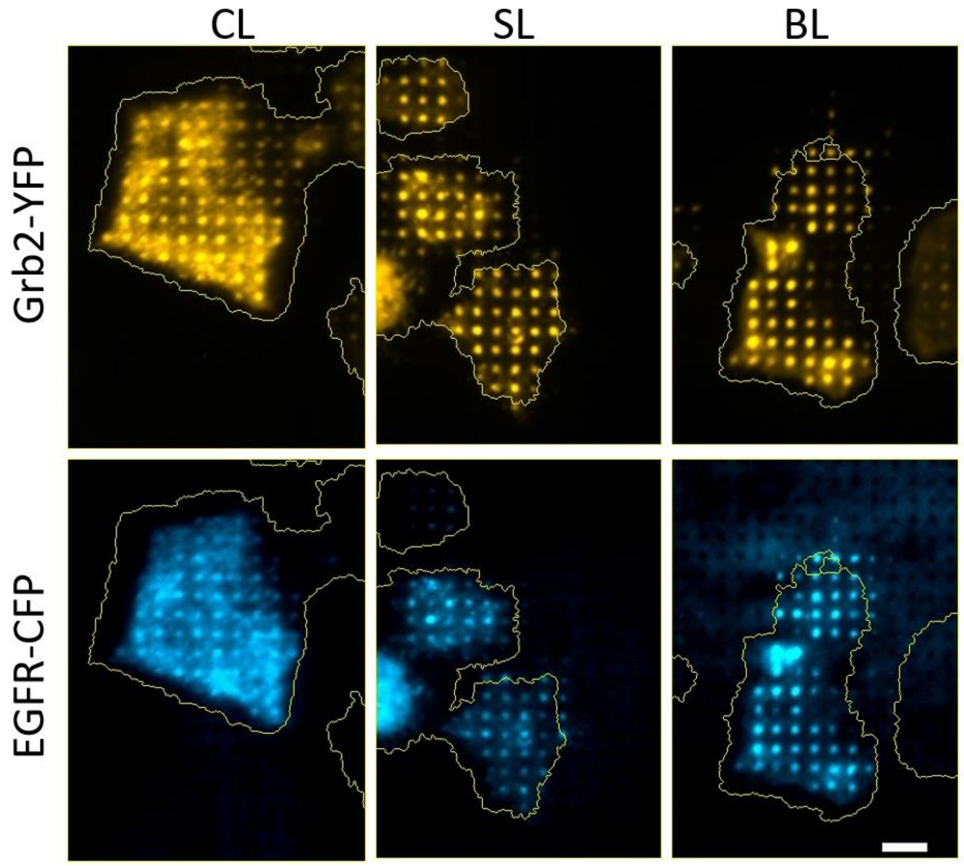

Figure S8. Representative TIRF microscopy images of HeLa cells transiently co-expressing EGFR-CFP and Grb2-YFP and grown on the different DON-micropatterned substrates shown in Figure 3B. Cell boundaries were detected by a watershed-based segmentation approach on raw images using the OpenCV library in Python version 3.9 in Spyder[7] and are depicted here to help the reader identify the cells. Scale bar: 5 µm.

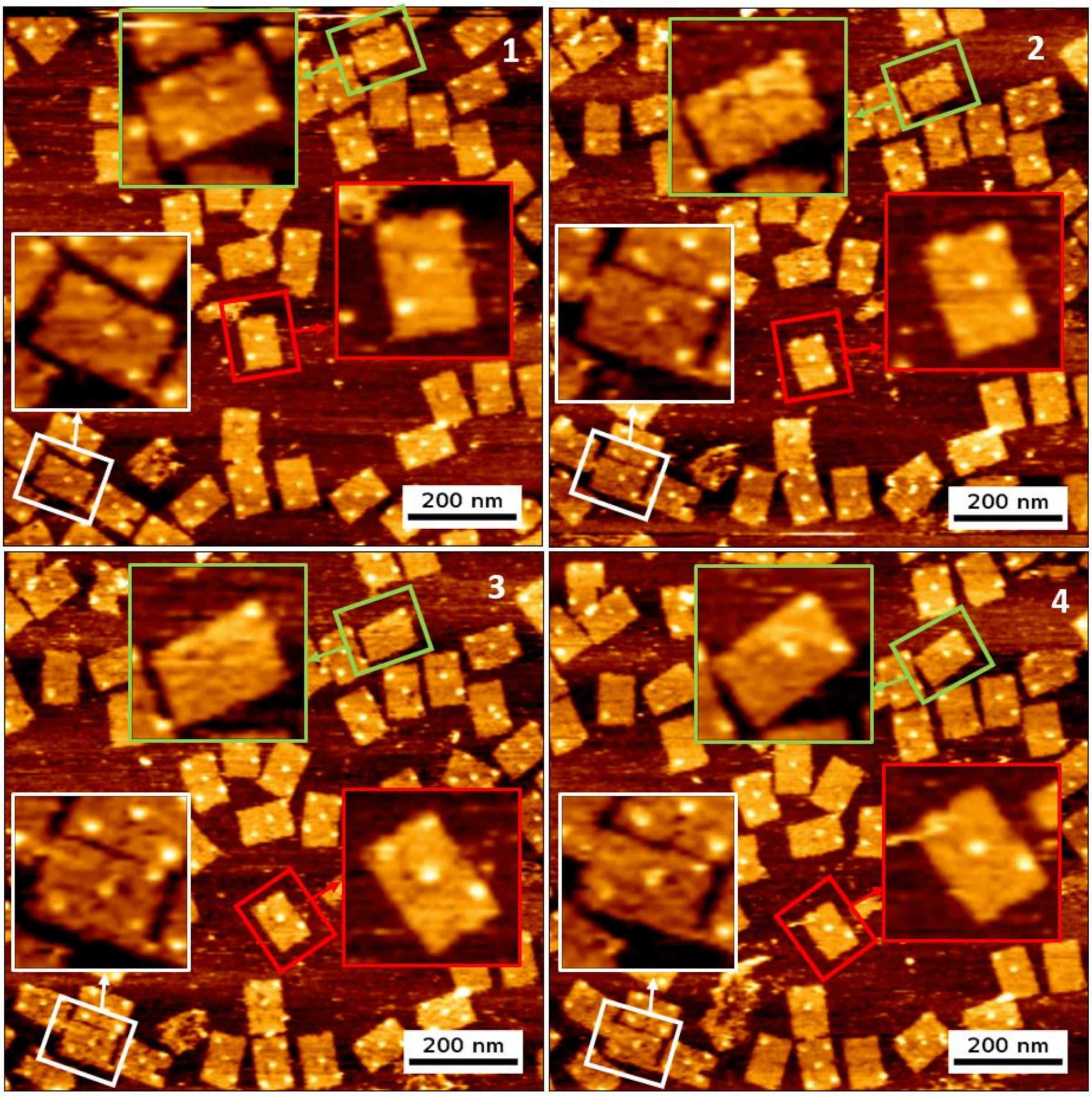

Figure S9. Standard AFM images (resolution 256 x 256 px^2, line rate 1 Hz), taken repeatedly from the same area using the CL-DON system. As shown in the selected enlarged examples, binding fluctuations can also be observed by this means. Note that takes about 4 minutes to scan each image, so the full measurement time was about 16 minutes. Due to the low temporal resolution, only sporadic fluctuations can be detected.

2. Supporting Movies

Supporting Movie 1_BL shows an example of BL-DON measured by HS-AFM in presence of STV.
Supporting Movie 2_SL shows an example of SL-DON measured by HS-AFM in presence of STV.
Supporting Movie 3_CL shows an example of CL-DON measured by HS-AFM in presence of STV.

3. Supporting Tables

Table S1. Unmodified staple strands used for the assembly of DON. The staples selected for modification of the lower or upper side of DON are indicated on the third column, as well as the modification and the position on the strand. “Lower” and “upper side” refers to the position on DON plane after binding on a surface.

Name	Sequence (5'-3')	Modification
3[256]-5[255]	GCCACCACGAAACCATCGATAGCAAAAGGGCG	
11[128]-13[127]	GCTATATTGCAAAATTAAGCAATACAGTCAAA	
19[128]-21[127]	ACTCGTCGAGGGCTTAAGCTACGTTGCGTTGC	
12[79]-10[80]	ATTATGACGAGTAGATTTAGTTTGTGAATATA	
7[320]-9[319]	AAGTAAGCTACAAAATAAACAGCCACGCTAAC	
19[192]-20[176]	GCAACAGTAGGCGGTCAGTATTAAAATCATTT	
16[47]-14[48]	CCTCAGGAATTTAAATTGTAAACGAGAAAAGC	
4[47]-2[48]	AGCCGGAACCTCAGCAGCGAAAGATTGCAGGG	
22[47]-20[48]	GTTTGCCCCAATTCCACACAACATGTCATAGC	
20[79]-18[80]	CCGAGCTCGCTCGCCCTGGAGTGATGGTTGTG	
3[192]-4[176]	TTCGGTCACTGTAGCGCGTTTTCAGAGGCAAA	
11[256]-13[255]	TAATGCAGAGTAGGGCTTAATTGAGTTAATTT	
19[320]-21[319]	GGAAGGTTGTAAGAATACGTGGCATCTGGCCA	
1[320]-3[319]	CTGAGACTAGAGCCGCCGCCAGCAGAGCCACC	
5[160]-7[159]	AAATCAACTGCCCTGACGAGAAACCATAGTAA	
13[320]-15[319]	AGAATAAATTTTCCCTTAGAATCCGAATAACC	
3[128]-5[127]	CATTAAACATACCAAGCGCGAAACTCAAGAGT	
10[47]-8[48]	ACTAAAGTGAGAATGACCATAAATTTGAATCC	
4[79]-2[80]	GTCGAAATGGTAGCAACGGCTACAACGCATAA	
19[256]-21[255]	TCAAATATAGCCCTAAAACATCGCTCTGAAAT	
6[79]-4[80]	GATTTTAAGACAGATGAACGGTGTTAAATTGT	
10[175]-9[191]	AACTCCAATCAAAGCGAACCAGACTTATCCGG	
14[47]-12[48]	CCCAAAAATTAGAACCCTCATATACGGGAGAA	
1[160]-3[159]	AAAAAAAATATCAGCTTGCTTTCGTGCCACTA	
7[192]-8[176]	CAAAGTCAGGAGAATTAACTGAACTTACCAGA	
11[304]-9[303]	GAAAAATATCCTTATCATTCCAATCCTGAA	
2[271]-0[272]	TATTCACAATGCCCCCTGCCTATTTGATATAA	
12[343]-13[343]	TTTTTTAATTACTAGACACCGGAATCATTTTT	
19[160]-21[159]	GAATCGGCTCTGACCTCCTGGTTGGGAAACCT	
1[224]-3[223]	GGGTCAGTAAGCGCAGTCTCTGAGTTTGCC	
3[304]-1[303]	CACCCTCATTGACAGGAGGTTGATGAAACA	
8[143]-6[144]	TGCAAAAAAAGGAATTACGAGGACCAGAAC	
4[271]-2[272]	TCACCAATCGGAACCGCCTCCCTCGGCCTTGA	
8[271]-6[272]	TTTAACGTAGAGCAAGAAACAATGGGCATGAT	
23[224]-22[240]	TTGCTTTGACGAGCACGTATAACGCATCACTT	
22[63]-23[71]	AAGCGGTCGGCAAAATCCCTTA	
0[327]-1[319]	AGCGGGGTTTTGCTCATTAAGAGG	
6[343]-7[343]	TTTTTCAAAGTTACCAAGATAGCCGAATTTTT	
16[271]-14[272]	TACCTTTTAATTACCTTTTTTAATGCTGAGAA	
17[224]-19[223]	CATCATATCTTTGCCCGAACGTTGCAGCAA	
10[343]-11[343]	TTTTTAGAAACCAATCTACGAGCATGTTTTTT	
5[256]-7[255]	ACATTCAACTTATTACGCAGTATGGTTAAGCC	

13[24]-12[24]	TTTTTGATAAAAATTTTTCAACGCAAGTTTTT
23[24]-22[24]	TTTTTAATCCTGTTTGCAGCAGGCGAATTTTT
13[128]-15[127]	TCACCATCAGTCTGGAGCAAACAAAACGCCAT
9[192]-10[176]	TATTCTAAATCAGATATAGAAGGCCGGAAGCA
18[111]-16[112]	AGCCAGGGGTTGGGAAGGGCGATCGGTCACGT
21[128]-23[127]	GCTCACTGGTTTTTCTTTTCACCAGAACAAGA
10[143]-8[144]	TAATTGCAGACTTCAAATATCGGAGGCTTT
17[128]-19[127]	CCTCTTCGCCAGTGCCAAGCTTTCTGTAAGCA
10[111]-8[112]	GCGGATGGATTGCATCAAAAAGATGGGTAATA
2[239]-0[240]	AGAATGGAGCCTTGAGTAACAGTGCCGTACTC
5[24]-4[24]	TTTTTCGGTCAATCATCGAGGCGCAGATTTTT
12[63]-14[64]	ACTTTTGTTTTAAATGCAATGCCCCCGGTT
0[271]-1[255]	GTATAGCCCGGAATAGGTGTATCACCCGTATA
14[239]-12[240]	CTGAGAGATTTCAAATATATTTTAGAATCGCC
23[72]-22[80]	TAAATCAAAAGAATAGGCCTGGCC
10[239]-8[240]	TTCATCGTTTGCGGGAGGTTTTGAAGCCTTTA
7[256]-9[255]	CAATAATACAAAAATGAAAATAGCAGCCTTAA
21[192]-22[176]	GCCATTGCATATCCAGAACAATATCGGCCAAC
6[271]-4[272]	TAAGACTCCCGATTGAGGGAGGGACGGAAACG
21[320]-23[327]	ACAGAGATTCAGTGAGGCCACCGAGGAACGGTACGCCAGA
14[111]-12[112]	ATGAACGGTGAGAAAGGCCGGAGAAAGCCTCA
12[143]-10[144]	AGAATTATTCATTTGGGGCGCGAGTACCTT
15[24]-14[24]	TTTTTTATAAGCAAATCAGGAAGATTGTTTTT
4[111]-2[112]	ACGGAGATAAGACTTTTTCATGAGAGTTGCGC
17[208]-15[207]	AAGGAGCGAGTTACAAAATCGCGCAAAAGAAG
21[224]-23[223]	TACCTACAAGAAGAACTCAAACTACTATGG
20[343]-21[343]	TTTTTGACCTGAAAGCAGAACCCTTCTTTTTT
7[24]-6[24]	TTTTTCTACGTTAATAGGAAGAAAAATTTTTT
14[79]-12[80]	CATATGTACTGAGTAATGTGTAGGCCAAAAAC
4[143]-2[144]	AGCGATTGGGTAAAATACGTAAAGGTGAAT
15[192]-16[176]	ACCTGAGCAGAGGCGAATTATTCAGAGCGAGT
18[175]-17[191]	CGCCAGGGCTGCAAGGCGATTAAGTTTGCGGA
12[111]-10[112]	GAGCATAACGCAAATGGTCAATAATCATTTTT
5[288]-7[287]	ATTGACGGAATACCCAAAAGAACTAAATAGCA
17[24]-16[24]	TTTTTCAGCCAGCTTTAGATCGCACTCTTTTT
10[79]-8[80]	ATGCTGTATACCCTGACTATTATATCCAATAC
13[96]-15[95]	CAAAAGGGTAATCGTAAAACTAGCTTGTTAAA
2[79]-0[72]	CCGATATACAACTTTCAACAGTTTAGCATTCCACAGACAG
0[111]-1[95]	ACCAGTACAAACTACAACGCCTGTCAGCGGAG
16[111]-14[112]	TGGTGTAGTTTTTTAACCAATAGGGAGAATCG
22[111]-20[112]	GGCAACAGGCTAACTCACATTAATGGTGCTTG
0[143]-1[127]	GAACCCATGTACCGTAACACTGAGGAATTGC
7[288]-9[287]	ATAGCTATATCCAAATAAGAAACGCCCAGCTA
21[96]-23[95]	ATGAGTGACTGATTGCCCTTCACCCCCGAGAT
21[256]-23[255]	GGATTATTCTTTGATTAGTAATAATGCTTTCC
6[111]-4[112]	TTCAACTTGGCTGGCTGACCTTCAAAAGTACA

9[208]-7[207]	GGCGTTTTGGAAGCGCATTAGACGGAGGGTAA
18[271]-16[272]	TATTAGACATAATCCTGATTGTTTAGTAACAG
23[128]-22[144]	GTCCACTATTAAAGAACGTGGACTTGGGCGC
6[143]-4[144]	GAGTAGTCAAGAACCGGATATTTGACCCCC
8[47]-6[48]	CCCTCAAAAAACGAACTAACGGAAACCAGTCA
8[79]-6[80]	TGCGGAATTAGAAAGATTCATCAGCCTTATGC
11[24]-10[24]	TTTTTAAGTTTCATTCACGGTGTCTGGTTTTT
8[111]-6[112]	GTAAAATGCCACATTCAACTAATGGGTTTAAT
9[256]-11[255]	ATCAAGATGAGAACAAGCAAGCCGTGTTCAGC
18[63]-20[64]	TTAAGTGTTCTAATCTATTTACGAATTCGT
22[143]-20[144]	CAGGGTGCCCGCTTTCCAGTCGGTGTAATG
1[208]-0[200]	TAATAAGTCAGAACCGCCACCCTC
3[320]-5[319]	ACCCTCAGATTAGAGCCAGCAAAAGGTGAATT
10[271]-8[272]	CACTCATCTAGTTGCTATTTTGCAATTTTTTG
21[160]-23[167]	GTCGTGCCAGAGGCGGTTTGCGTATCCAACGTCAAAGGGC
15[96]-17[95]	TCAGCTCAATGGGCGCATCGTAACTTCAGGCT
20[47]-18[48]	TGTTTCCTGCCAAAATAACCCCGCTCCTTAGT
4[239]-2[240]	ATCAGTAGATAATCAAAATCACCGTTAAAGCC
11[320]-13[319]	TCCTAATTAAAAGCCTGTTTAGTAGTTAAATA
15[304]-13[303]	ATGTGAGTTTGAAAACATAGCGATGTGATA
13[224]-15[223]	GAAAACTTCTACCTTTTTAACCTACAAACA
0[343]-1[343]	TTTTTGGATTAGGATTCCTCAAGAGAATTTTT
13[160]-15[159]	CTGATAAAATCTACAAAGGCTATCTCCTGTAG
22[343]-23[343]	TTTTTTGTTTTTATAAATCCTGAGAAGTTTTT
14[63]-16[64]	GATAATCTTAATATTTTGTTAATGAGGGGA
15[224]-17[223]	TCAAGAAATTGCTTTGAATACCAGAATTAT
23[96]-22[112]	AGGGTTGAGTGTTGTTCCAGTTTGGTGAGACG
20[271]-18[272]	GAACTGATCAAACCCTCAATCAATTTTAGAAG
8[343]-9[343]	TTTTTAATTTGCCAGTTTCCAGAGCCTTTTTT
17[304]-15[303]	AGGGTTAGGATTTTCAGGTTTAATCAATAT
18[143]-16[144]	ACGACGGCTATTACGCCAGCTGGAACAAAC
23[40]-22[48]	ATGGTGGTTCCGAAATCCACGCTG
11[208]-9[207]	GGTAAAGTGCCCAATAGCAAGCAAGAACGCGA
16[143]-14[144]	GGCGGATATTCGCGTCTGGCCTAGGTCATT
18[79]-16[80]	AATTCATGAAAGCGCCATTCGCCACGTGCATC
19[24]-18[24]	TTTTTGACAATGTCCCGTCAACCTTATTTTTT
20[143]-18[144]	AGTAAACGTGGGCACGAATATAGTTGTAAA
0[71]-2[64]	CCCTCATAGTTAGCGTGGGATTTTGCTAAATTCGGTCG
16[79]-14[80]	TGCCAGTTAATTCGCATTAAATTTATGTCAAT
14[271]-12[272]	GAGTCAATGACCTAAATTTAATGGAAAGCCAA
23[256]-22[272]	TCGTTAGAATCAGAGCGGGAGCTACGTTGTAG
9[320]-11[319]	GAGCGTCTAATAATCGGCTGTCTTATATCCCA
5[192]-6[176]	GTTTATTTAAACGCAAAGACACCATTCAGTGA
2[143]-0[144]	TTCTTAAATTTTTTCACGTTGACCCAATAG
6[239]-4[240]	CGTAGAAAACCAGCGCCAAAGACAGCACCGTA
14[143]-12[144]	GCCTGAGAATATGATATTCAACCAAGGCAA

9[96]-11[95]	CAAAGCGGCTTAGAGCTTAATTGCACCATTAG
21[24]-20[24]	TTTTTTTATCCGCTCAGTGTGAAATTGTTTTT
18[47]-16[48]	GCTGAATTCCGGCACCGCTTCTGGAGTATCGG
9[304]-7[303]	TCTTACCAATATTATTTATCCCACTTACCG
15[320]-17[319]	TTGCTTCTATAAAGAAATTGCGTAAACCTACC
5[128]-7[127]	AATCTTGAAAATTGGGCTTGAGATCAGATACA
1[256]-3[255]	AACAGTTAAACAAATAAATCCTCAGAACCAGA
2[63]-4[64]	CTGAGGCCAGCATCGGAACGAGCCGCGACC
15[160]-17[159]	CCAGCTTTGTCGGATTCTCCGTGGGCGAAAGG
22[175]-21[191]	GCGCGGGGAGCTGCATTAATGAATTACCGCCA
21[288]-23[295]	ACGACCAGCATCACGCAAATTAACAACAGGAGGCCGATTA
22[79]-20[80]	CTGAGAGAAAAGTGTAAAGCCTGGCCCGGGTA
13[192]-14[176]	TGCAAATCTATATAACTATATGTATAGCTATT
5[304]-3[303]	TCATTAAATCACCAGTAGCACCAGAACCGC
21[208]-19[207]	AAACGCTCAGATAAAACAGAGGTGGCCACGCT
20[175]-19[191]	CTCCGAACTGACGCATTTCACATACACCGCCT
8[63]-10[64]	ATATTCACAAAAATCAGGTCTTGCTCAACA
16[239]-14[240]	TCGCCTGAACAAAATTAATTACATTCATAGGT
13[256]-15[255]	CATCTTCTAGTGAATTTATCAAAATTAACAAT
21[304]-19[303]	AGGGACATCAGACAATATTTTTGAATCAAC
5[224]-7[223]	TATGGTTTATACATACATAAAGGTAATATC
20[63]-22[64]	AATCATGACGAGCCGGAAGCATGTTGCAGC
22[239]-20[240]	GCCTGAGTTTTTGACGCTCAATCGCATTAAAA
2[47]-0[40]	AGTTAAAGAAATGAATTTTCTGTATAACGATCTAAAGTTT
1[128]-3[127]	GAATAATAACAGCTTGATACCGATGAAGTTTC
20[111]-18[112]	TTACCTCGGTGCGGCCCTGCCATCTCAGGAGA
22[271]-20[272]	CAATACTTTACATTGGCAGATTCATAATGCGC
23[200]-21[207]	CCGCTACAGGGCGCGTATCGGCCTTGCTGGTAAACAGGAA
23[168]-23[199]	GAAAAACCGTCTATCACGCCGCGCTTAATGCG
6[47]-4[48]	GGACGTTGAAGGGAACCGAACTGATGTTACTT
15[256]-17[255]	TTCATTTGACATCGGGAGAAACAAATGGCAAT
3[24]-2[24]	TTTTTGGGATCGTCACGCCGCTTTTGCTTTTT
8[175]-7[191]	CGACGATACTATCATAACCCTCGTACCCTGAA
17[160]-19[159]	GGGATGTGTTTTCCCAGTCACGACGGGGCCTT
9[128]-11[127]	AGCCCGAATCCTTTTGATAAGAGGCCTGTTTA
5[320]-7[319]	ATCACCGTGAAGGAAACCGAGGAATTTAAGAA
7[128]-9[127]	TAACGCCAGAAGTTTTGCCAGAGGTAAGAGGA
1[24]-0[24]	TTTTTCAGACGTTAGTTGTCGTCTTTCTTTTT
14[343]-15[343]	TTTTTCTATTAATTAAGTAAATCGTCGTTTTT
15[288]-17[287]	TACATAAACGTCAGATGAATATACGGATTATA
2[175]-1[191]	TATCGGTTGGCTCCAAAAGGAGCCTTTGATGA
17[256]-19[255]	TCATCAATTTTACAAACAATTCGAGCTGAACC
12[47]-10[48]	GCCTTTATCATATAACAGTTGATTAATATGCA
1[304]-0[296]	TGAAAGTAGTACCAGGCGGATAA
3[208]-1[207]	TTATTAGCATTTACCGTTCCAGTATGTACTGG
8[239]-6[240]	CAGAGAGAAACCCACAAGAATTGATTAGCAAA

2[343]-3[343]	TTTTTGAACCACCACCAGCCGCCACCATTTTT	
6[175]-5[191]	ATAAGGCTGTAACAAAGCTGCTCACGGAATAA	
15[208]-13[207]	ATGATGAACCGGCTTAGGTTGGGTCAATCGCA	
18[239]-16[240]	ATTAAATCTCCTGATTATCAGATGTAACGGAT	
5[208]-3[207]	TCAATAGATGCCTTTAGCGTCAGATAGCCCCC	
15[128]-17[127]	CAAAAATATGACCGTAATGGGATAGGTGCGGG	
12[271]-10[272]	CGCTCAACAACGCGCCTGTTTATCAAGTACCG	
17[192]-18[176]	ACAAAGAATGAGTAACATTATCATTTGGGTAA	
0[175]-1[159]	ACCCTCATTTTCAGGGATAGCAAGAAATCTCC	
4[343]-5[343]	TTTTTGCCATTTGGGACACCGACTTGATTTTT	
17[320]-19[319]	ATATCAAACTAACAACTAATAGATGGAATTGA	
16[343]-17[343]	TTTTTGTAAAACAGAAATTATTTGCACTTTTT	
9[24]-8[24]	TTTTTGTTCAGAAAACTGCTTTAAACATTTTT	
18[343]-19[343]	TTTTTCTTTAGGAGCAATCTAAAATATTTTTT	
1[192]-2[176]	TACAGGAGAGCGTCATACATGGCTTTTAATTG	
12[239]-10[240]	ATATTTAAACGACAATAAACAACATTTTTATT	
4[175]-3[191]	AGAATACACCAACCTAAAACGAAATCGGCATT	
6[63]-8[64]	TCATTATCAACATTATTACAGGCGTCATAA	
14[175]-13[191]	TTTGAGAGTTAATGCCGGAGAGGGAATGCTGA	
23[296]-21[303]	AAGGGATTTTAGACAGTAAAAGAGTCTGTCTAATAAA	
9[224]-11[223]	CTCCCGACAGGAATCATTACCGCAATTCTG	
0[295]-1[287]	GTGCCGTCGAGAGGGTTCGGAACC	
0[239]-1[223]	AGGAGGTTTAGTACCGCCACCCTTTTAACG	
20[239]-18[240]	ATACCGAATCTAAAGCATCACCTTCAACTCGT	
2[111]-0[112]	CGACAATGGAAAGGAACAACTAAAGTTTCGTC	
9[288]-11[287]	CAATTTTAGAACGGGTATTAAACCAACAATAG	
0[199]-0[176]	AGAACCGCCACCCTCAGAGCCACC	
9[160]-11[159]	TTCGAGCTCAGGTCAGGATTAGAGAGCTGAAA	
16[175]-15[191]	AACAACCCCATCAACATTAAATGTTTTCAATT	
13[288]-15[287]	ACCGACCGTAGCTTAGATTAAGACGGAAACAG	
7[160]-9[159]	GAGCAACAAAAACCAAAATAGCGACGTTTTAA	
11[224]-13[223]	TCCAGACGCAACGCCAACATGTAAACGCGA	
3[224]-5[223]	ATCTTTTCCGACAGAATCAAGTTAAATTCA	
5[96]-7[95]	GGCGCATATAATCATTGTGAATTATTGAGATT	
19[224]-21[223]	ATGAAAAACGAACCACCAGCAGAATGGAAA	
11[96]-13[95]	ATACATTTAGCTAAATCGGTTGTATAAAGATT	
11[160]-13[159]	AGGTGGCACAATAAATCATACAGGCGTTCTAG	
11[288]-13[287]	ATAAGTCCAAATTCTTACCAGTATTTTGAAAT	
7[96]-9[95]	TAGGAATATTTAGACTGGATAGCGGTCAGAAG	
7[224]-9[223]	AGAGAGATATAACATAAAAACAGAGCGAAC	
3[160]-5[159]	CGAAGGCACTAAAACACTCATCTTCATTACCC	
19[304]-17[303]	AGTTGAAATAGAGCCGTCAATAGTAATGGA	Lower side (3'protruding)
13[208]-11[207]	AGACAAAGATTTAGGCAGAGGCATCGACAAAA	Lower side (3'protruding)
16[63]-18[64]	CGACGACTGCCGGAAACCAGGCCGCACGAC	Lower side (3'protruding)

13[304]-11[303]	AATAAGGCTCATATGCGTTATACTGAACAA	Lower side (3'protruding)
10[63]-12[64]	TGTTTTACCCAATTCTGCGAACCCTGTAAT	Lower side (3'protruding)
4[63]-6[64]	TGCTCCACCAACTTTGAAAGAGGAACTGGC	Lower side (3'protruding)
19[208]-17[207]	GAGAGCCAATTAATTTTAAAAGTTACCACCAG	Lower side (3' protruding)
7[304]-5[303]	AAGCCCTTACGCAATAATAACGGAAATTAT	Lower side (3'protruding)
7[208]-5[207]	TTGAGCGCTGGCAACATATAAAAGTGTCACAA	Lower side (3'protruding)
3[96]-5[95]	GAGGACTATTGTATCATCGCCTGAACAGACCA	Upper side (5'biotin)
19[96]-21[95]	TACCGACAATAAAGACGGAGGATCGGTGCCTA	Upper side (5'biotin)
11[192]-12[176]	TAAAGTACTTTCGAGCCAGTAATATAGTAGCA	Upper side (5'biotin)
3[288]-5[287]	CACCCTCATTACCATTAGCAAGGCAGGTAAAT	Upper side (5'biotin)
19[288]-21[287]	AGTTGGCAAATGGCTATTAGTCTTCCAGTCAC	Upper side (5'biotin)
1[96]-3[95]	TGAGAATAACAACAACCATCGCCCGAGGCTTT	Upper side (3'biotinTg)
17[96]-19[95]	GCGCAACTTGGATGTTCTTCTAAGCTCTATGA	Upper side (3'biotinTg)
1[288]-3[287]	TATTATTCGGCAGGTCAGACGATTAGAGCCGC	Upper side (3'biotinTg)
17[288]-19[287]	CTTCTGAAATAATACATTTGAGGAATCTGGTC	Upper side (3'biotinTg)
12[175]-11[191]	TTAACATCTCAATTCTACTAATAGAGAGAATA	Upper side (3'biotinTg)

Table S2. Modified staple strands used for the assembly of DONs. The detailed function is indicated on the third column.

Name	Sequence (5'-3')	Function
19[304]-17[303]-cTr1	AGTTGAAATAGAGCCGTCAATAGTAATGGA<br>TTTTTTTTCATCATCATCATCATCATCATCAT	3' Protruding – Single-stranded binding tag
13[208]-11[207]-cTr1	AGACAAAGATTTAGGCAGAGGCATCGACAAAA<br>TTTTTTTTCATCATCATCATCATCATCATCAT	3' Protruding – Single-stranded binding tag
16[63]-18[64]-cTr1	CGACGACTGCCGGAAACCAGGCCGCACGAC<br>TTTTTTTTCATCATCATCATCATCATCATCAT	3' Protruding – Single-stranded binding tag
13[304]-11[303]-cTr1	AATAAGGCTCATATGCGTTATACTGAACAA<br>TTTTTTTTCATCATCATCATCATCATCATCAT	3' Protruding – Single-stranded binding tag
10[63]-12[64]-cTr1	TGTTTTACCCAATTCTGCGAACCCTGTAA<br>TTTTTTTTTCATCATCATCATCATCATCATCAT	3' Protruding – Single-stranded binding tag
4[63]-6[64]-cTr1	TGCTCCACCAACTTTGAAAGAGGAACTGGC<br>TTTTTTTTCATCATCATCATCATCATCATCAT	3' Protruding – Single-stranded binding tag
19[208]-17[207]-cTr1	GAGAGCCAATTAATTTTAAAAGTTACCACCAG<br>TTTTTTTTCATCATCATCATCATCATCATCAT	3' Protruding – Single-stranded binding tag
7[304]-5[303]-cTr1	AAGCCCTTACGCAATAATAACGGAAATTA<br>TTTTTTTTTCATCATCATCATCATCATCATCAT	3' Protruding – Single-stranded binding tag
7[208]-5[207]-cTr1	TTGAGCGCTGGCAACATATAAAAGTGTCACAA<br>TTTTTTTTCATCATCATCATCATCATCATCAT	3' Protruding – Single-stranded binding tag
3[96]-5[95]-BioC	[Btn]GAGGACTATTGTATCATCGCCTGAACAGACCA	5'biotin – Biotin site in CL-DON
19[96]-21[95]-BioC	[Btn]TACCGACAATAAAGACGGAGGATCGGTGCCTA	5'biotin – Biotin site in CL-DON
11[192]-12[176]-BioC	[Btn]TAAAGTACTTTCGAGCCAGTAATATAGTAGCA	5'biotin – Biotin site in CL-DON
3[288]-5[287]-BioC	[Btn]CACCCTCATTACCATTAGCAAGGCAGGTAAAT	5'biotin – Biotin site in CL-DON
19[288]-21[287]-BioC	[Btn]AGTTGGCAAATGGCTATTAGTCTTCCAGTCAC	5'biotin – Biotin site in CL-DON
3[96]-5[95]-BioS	[Btn]TTTTTTTGAGGACTATTGTATCATCGCCTGAAC<br>AGACCA	5'biotin – Biotin site in SL- and BL-DON
19[96]-21[95]-BioS	[Btn]TTTTTTTTACCGACAATAAAGACGGAGGATCGG<br>TGCCTA	5'biotin – Biotin site in SL- and BL-DON
11[192]-12[176]-BioS	[Btn]TTTTTTTTAAAGTACTTTCGAGCCAGTAATATA<br>GTAGCA	5'biotin – Biotin site in SL- and BL-DON
3[288]-5[287]-BioS	[Btn]TTTTTTTCACCCTCATTACCATTAGCAAGGCAG<br>GTAAAT	5'biotin – Biotin site in SL- and BL-DON
19[288]-21[287]-BioS	[Btn]TTTTTTTAGTTGGCAAATGGCTATTAGTCTTCC<br>AGTCAC	5'biotin – Biotin site in SL- and BL-DON
12[175]-11[191]-BioB	TTAACATCTCAATTCTACTAATAGAGAGAATATTTT<br>TTT[BtnTg]	3'biotin – Biotin site in BL-DON
1[96]-3[95]-BioB	TGAGAATAACAACAACCATCGCCCGAGGCTTTTTTT<br>TTT[BtnTg]	3'biotin – Biotin site in BL-DON
1[288]-3[287]- BioB	TATTATTCGGCAGGTCAGACGATTAGAGCCGCTTTT<br>TTT[BtnTg]	3'biotin – Biotin site in BL-DON
17[288]-19[287]-BioB	CTTCTGAAATAATACATTTGAGGAATCTGGTCTTTT<br>TTT[BtnTg]	3'biotin – Biotin site in BL-DON
17[96]-19[95]- BioB	GCGCAACTTGGATGTTCTTCTAAGCTCTATGATTTT<br>TTT[BtnTg]	3'biotin – Biotin site in BL-DON

Table S3. AFM techniques used for characterization of processes on DNA origami nanostructures.1)

	Line Rate (Hz)	Resolution (px^2)	Time per frame (s)	Scan Rate (fps)	Reference
**Conventional AFM**	2	360 x 360	180	0.006	[8]
**Fast–scan/ High–speed AFM**	ns	ns	10	0.1	[9]
	70	512 x 512	7	0.1	[6]
	ns	ns	5	0.2	[10]
	ns	ns	1	1	[11]
	ns	ns	1	1	[12]
	ns	ns	0.5	2	[13]
	2000	100 x 100	0.05	20 2)	this work

1) To achieve optimal spatiotemporal resolution in the analysis of STV-biotinylated DON binding, HS-AFM was employed in this study. Improvements in the temporal resolution of AFM over the past decades have pushed the limits of the scanning rates to 0.2 – 20 frames per second (fps), so that acquisition times in the range of 5 – 0.05 s per frame are currently feasible, enabling the dynamic analysis of biological samples.[14] High-speed AFM has previously been used for the investigation of conformational changes of DNA or protein binding on DON platforms,[6, 9-11, 13] however, some measurement parameters were not specified (ns). In this work a scan rate of 20 fps was applied for the first time to investigate binding dynamics between STV and DNA nanostructures, using the NanoRacer HS-AFM system (JPK BioAFM, Bruker, Germany).

2) Resolution = # Horizontal lines x # Vertical lines;
Time per frame (s) = # Horizontal Lines/Line Rate (s^{-1});
Scan Rate (fps) = 1/Time per frame (s).

Table S4. Medians, interquartile ranges (IQR) and *P* values for pairwise comparisons obtained for the analysis of R, t_{on} and t_{off} data. The statistical test performed was 1-way ANOVA Kruskal Wallis. Significance values have been adjusted by the Dunn-Bonferroni correction for multiple tests. Interquartile ranges (IQR) are given as Q1 to Q3. Note that for the determination of outliers, IQR are calculated by subtracting Q3 – Q1. Graphical representations are shown in Figure 2D, E and F.

	**DON**	**Median (%)**	**IQR (%)**	**DON 1 – DON 2**	***P* value**
R	CL	16.40	0.10 – 93.66	CL – SL	0.021
	SL	60.83	30.46 – 93.91	CL – BL	0.800
	BL	100	97.39 – 100	SL – BL	0.023

	**DON**	**Median (s)**	**IQR (s)**	**DON 1 – DON 2**	***P* value**
t_{on}	CL	0.60	0.15 – 2.5	CL – SL	0.021
	SL	0.75	0.25 – 2.78	CL – BL	0.800
	BL	0.45	0.15 – 1.80	SL – BL	0.023

	**DON**	**Median (s)**	**IQR (s)**	**DON 1 – DON 2**	***P* value**
t_{off}	CL	0.25	0.10 – 0.60	CL – SL	<.001
	SL	0.53	0.15 – 1.40	CL – BL	<.001
	BL	0.15	0.25 – 1.20	SL – BL	0.252

4. References

[1] S. M. Douglas, A. H. Marblestone, S. Teerapittayanon, A. Vazquez, G. M. Church, W. M. Shih, *Nucleic acids research* **2009**, *37*, 5001-5006.

[2] U. Diwekar, A. David, in *BONUS Algorithm for Large Scale Stochastic Nonlinear Programming Problems*, Springer, **2015**, pp. 27-34.

[3] a) R. Tapia-Rojo, E. C. Eckels, J. M. Fernández, *Proceedings of the National Academy of Sciences* **2019**, *116*, 7873-7878; b) R. A. Rosales, W. J. Fitzgerald, S. B. Hladky, *Biophysical journal* **2002**, *82*, 29-35.

[4] M. Hubert, S. Van der Veeken, *Journal of Chemometrics: A Journal of the Chemometrics Society* **2008**, *22*, 235-246.

[5] M. Hubert, E. Vandervieren, *Computational statistics & data analysis* **2008**, *52*, 5186-5201.

[6] C. Kielar, S. Zhu, G. Grundmeier, A. Keller, *Angewandte Chemie* **2020**, *132*, 14442-14447.

[7] A. Mordvintsev, K. Abid, *https://media.readthedocs.org/pdf/opencv-python-tutroals/latest/opencv-python-tutroals.pdf*, **2014**.

[8] N. Wu, X. Zhou, D. M. Czajkowsky, M. Ye, D. Zeng, Y. Fu, C. Fan, J. Hu, B. Li, *Nanoscale* **2011**, *3*, 2481-2484.

[9] S. F. Wickham, M. Endo, Y. Katsuda, K. Hidaka, J. Bath, H. Sugiyama, A. J. Turberfield, *Nature nanotechnology* **2011**, *6*, 166-169.

[10] Y. Sannohe, M. Endo, Y. Katsuda, K. Hidaka, H. Sugiyama, *Journal of the American Chemical Society* **2010**, *132*, 16311-16313.

[11] M. Endo, Y. Katsuda, K. Hidaka, H. Sugiyama, *Angewandte Chemie International Edition* **2010**, *49*, 9412-9416.

[12] M. Endo, Y. Katsuda, K. Hidaka, H. Sugiyama, *Journal of the American Chemical Society* **2010**, *132*, 1592-1597.

[13] P. Zhang, X. Liu, P. Liu, F. Wang, H. Ariyama, T. Ando, J. Lin, L. Wang, J. Hu, B. Li, *Nature communications* **2020**, *11*, 1-9.

[14] a) T. Ando, *Current opinion in structural biology* **2014**, *28*, 63-68; b) A. Rajendran, M. Endo, H. Sugiyama, *Chemical reviews* **2014**, *114*, 1493-1520.

5. Author Contributions

C.M.D, H.H, K.S.R and C.M.N designed the project and conceived of the presented idea. C.M.D designed and synthetized the DON, and performed conventional AFM measurements and HS-AFM data analysis. M.G.C and A.I contributed to the HS-AFM data analysis. A.K and H.H performed the HS-AFM measurements. U.M and P.L performed the cell experiments. K.G contributed to the cell experiments. C.M.D and C.M.N wrote the manuscript. All authors discussed the results and contributed to the final manuscript.
